# Methodology for Cross-Talk Elimination in Simultaneous Voltage and Calcium Optical Mapping Measurements With Semasbestic Wavelengths

**DOI:** 10.3389/fphys.2022.812968

**Published:** 2022-02-11

**Authors:** Ilija Uzelac, Christopher J. Crowley, Shahriar Iravanian, Tae Yun Kim, Hee Cheol Cho, Flavio H. Fenton

**Affiliations:** ^1^School of Physics, Georgia Institute of Technology, Atlanta, GA, United States; ^2^Division of Cardiology, Section of Electrophysiology, Emory University Hospital, Atlanta, GA, United States; ^3^Department of Pediatrics, Emory University School of Medicine, Atlanta, GA, United States; ^4^Department of Biomedical Engineering, Emory University School of Medicine, Atlanta, GA, United States; ^5^The Sibley Heart Center, Children's Healthcare of Atlanta, Atlanta, GA, United States

**Keywords:** optical mapping, semasbestic wavelength, isosbestic point, fluorescent dyes, transmembrane voltage, intracellular free calcium concentration, alternans

## Abstract

Most cardiac arrhythmias at the whole heart level result from alteration of cell membrane ionic channels and intracellular calcium concentration ([Ca^2+^]_*i*_) cycling with emerging spatiotemporal behavior through tissue-level coupling. For example, dynamically induced spatial dispersion of action potential duration, QT prolongation, and alternans are clinical markers for arrhythmia susceptibility in regular and heart-failure patients that originate due to changes of the transmembrane voltage (*V*_m_) and [Ca^2+^]_*i*_. We present an optical-mapping methodology that permits simultaneous measurements of the *V*_m_ - [Ca^2+^]_*i*_ signals using a single-camera without cross-talk, allowing quantitative characterization of favorable/adverse cell and tissue dynamical effects occurring from remodeling and/or drugs in heart failure. We demonstrate theoretically and experimentally in six different species the existence of a family of excitation wavelengths, we termed semasbestic, that give no change in signal for one dye, and thus can be used to record signals from another dye, guaranteeing zero cross-talk.

## 1. Introduction

Heart failure (HF) is a global epidemic, affecting more than 64 million people worldwide (James et al., 2018) and is increasing in prevalence. In the US, about 6.9 million people have been diagnosed with HF, with an expected 24% increase to nearly 8.5 million by 2030 (Benjamin et al., 2018). The prognosis is poor: 20% die within 1 year and 80% within 8 years, resulting in over 655,000 deaths annually (Virani et al., 2020) in the US alone. More than half of HF deaths are due to ventricular fibrillation (Packer, 1985), and despite decades of study, the mechanisms by which HF predisposes patients to these ventricular arrhythmias are not well-understood. As a result, few treatment options are available. It is, therefore, crucial to identify how HF leads to the development of life-threatening cardiac arrhythmias. Although it is well-known that fibrosis and myocardial ischemia (Tomaselli and Zipes, 2004) can cause conduction abnormalities and cardiac arrhythmias, there is growing recognition that abnormal intracellular calcium cycling plays a fundamental role in the pathology of HF (Hoeker et al., 2009; Aistrup et al., 2011). Many studies have shown that disruptions in intracellular calcium concentration ([Ca^2+^]_*i*_) cycling, along with the complex voltage-calcium bidirectional coupling, can lead to action potential (AP) repolarization abnormalities that promote arrhythmias (Balijepalli and Kamp, 2008, 2011; Hoeker et al., 2009; Louch et al., 2010; Aistrup et al., 2011). This necessitates the development of effective methods that can investigate simultaneously the dynamics of the cell's transmembrane voltage (*V*_m_) and [Ca^2+^]_*i*_ in cardiac tissue.

**Figure 1:**
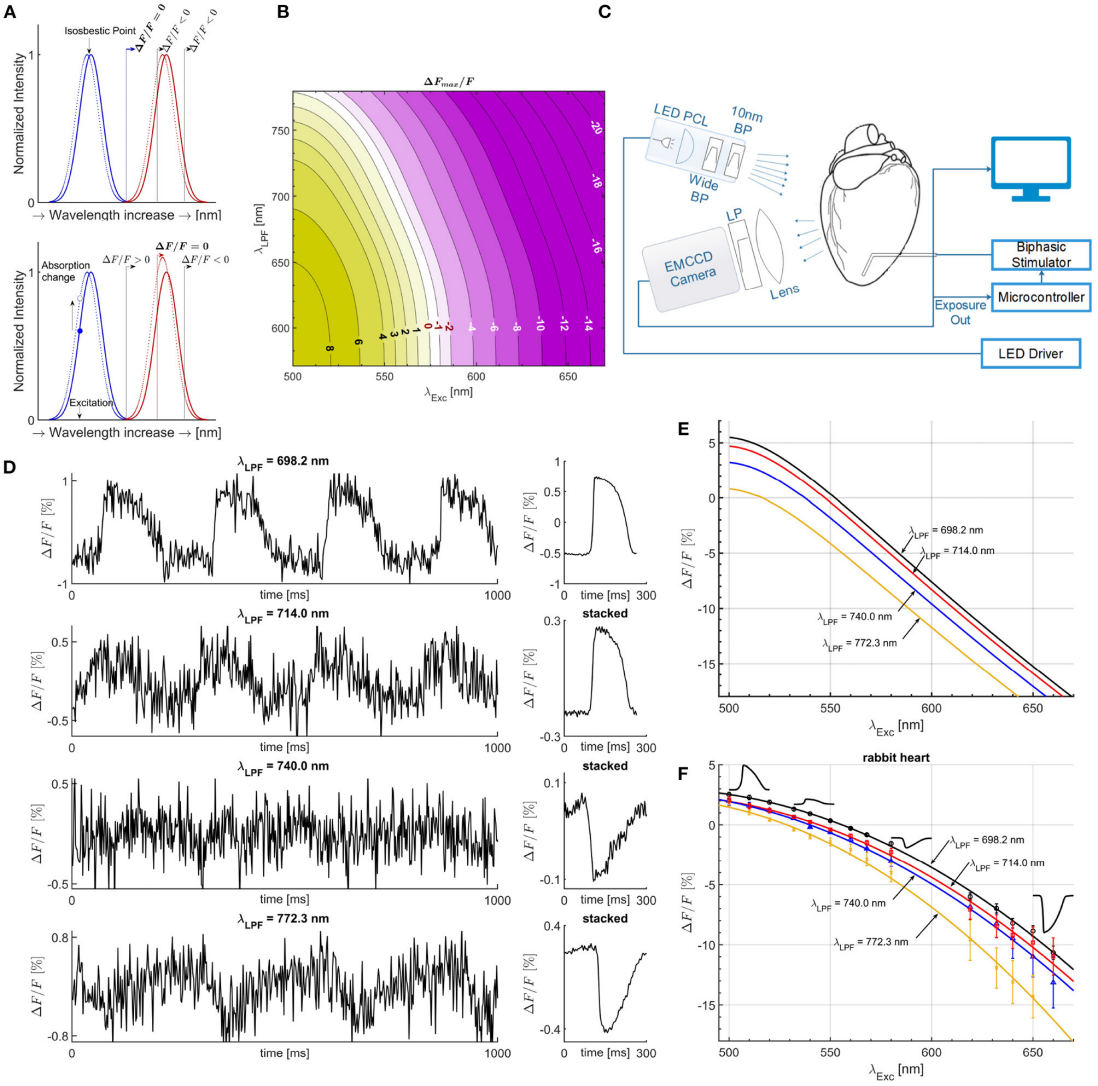
Mechanisms of measurement with an electrochromic *V*_m_ dye. **(A)** The Gaussian absorption (blue) and emission curves (red) are good fits for a given electrochromic *V*_m_ dye for illustration purposes, with solid lines for polarized membrane and leftward shifted dotted lines for maximally depolarized membrane by a propagating AP. For excitation at the *isosbestic* point and for a given LPF on the camera side passing only part of the emission spectra, as illustrated with the solid (black) vertical lines, Δ*F*/*F* is always negative. Δ*F*/*F* = 0 can only be achieved if the entire emission spectrum is obtained (solid blue vertical line), thereby not dependent on the optical setup, the λ_LPF_ value. Excitation left of the *isosbestic* point results in an amplitude increase of the emission spectra due to absorption coefficient increase with the shift of the absorption spectra. Depending on the filter λ_LPF_, overall Δ*F*/*F* sign can be negative or positive, and in between, there is a particular λ_LPF_ such that positive change cancels the negative change resulting in Δ*F*/*F* = 0, for specific λ_Exc_, termed *semasbestic* wavelength. **(B)** Theoretically calculated map of Δ*F*/*F* magnitude values as a function of λ_Exc_ and λ_LPF_, showing the transition from positive to negative Δ*F*/*F*, and continuous line of *semasbestic* wavelengths, Δ*F*/*F* = 0 isochrone line, for each λ_Exc_ - λ_LPF_ pair. **(C)** Illustration of the experimental setup. **(D)** APs from optical mapping measurements on isolated rabbit heart near a *semasbestic* wavelength. A 10 nm wide BP excitation filter was used of 540 nm nominal center wavelength along different LPFs on the camera's side. Due to low Δ*F*/*F* values SNR is low. However, ensemble averaging (stacking) increases SNR without filtering in post-processing. **(E)** Quadratic fit curves from Δ*F*/*F* simulated values **(B)**, for four different LPFs of the same λ_LPF_ values as LPF used in Δ*F*/*F* measurements on isolated hearts. **(F)** Quadratic fit curves from Δ*F*/*F* magnitude values for four different LPFs. Optical mapping recordings were performed on isolated rabbit heart for across a wide range of excitation wavelengths, from 500 to 660 nm. Zero crossings correspond to the *semasbestic* wavelengths. All λ_LPF_ values of LPFs are experimentally measured.

Optical mapping, developed in the mid-1970s, is the perfect methodology for the study of *V*_m_ -[Ca^2+^]_*i*_ in cardiac electrophysiology due to a high spatial and temporal resolutions. In essence the method originally consisted in measuring changes in *V*_m_ from changes in fluorescence intensity using *V*_m_ sensitive dyes. For example, electrochromic *V*_m_ dyes bind to the cell's membrane, and their absorption and emission spectra blue-shifts a few nanometers as the cell membrane depolarizes. By blocking the excitation light and part of the emission spectra with a long-pass-filter (LPF) placed over an electro-optical sensor (camera), fluorescence intensity is measured in practice (Figure 1). For small spectra shifts, the normalized change in fluorescence intensity (Δ*F*/*F*) is given by (F - F_0_) / F_0_, where F_0_ is the fluorescence intensity when the cell's membrane is polarized (the resting membrane potential), and F when the cell's membrane is depolarized (any other transmembrane potential) which to first approximation it is linearly proportional to the cell's transmembrane potential and thus closely reproduce the action potential (Figure 1D). With unprecedented high spatial and temporal resolution, optical mapping with *V*_m_ sensitive dyes have been used to characterize wave propagation across the tissue surface (Barone et al., 2020) and depth (Kelly et al., 2013), during regional ischemia (Sidorov et al., 2011) and provide evidence of reentrant waves as mechanisms of lethal arrhythmias such as ventricular tachycardia and fibrillation (Davidenko et al., 1992; Gray et al., 1998; Cherry and Fenton, 2008) that affect the electro-mechanical coupling (Christoph et al., 2018). Additionally, invasive and non-invasive techniques can also be characterized using optical mapping, such radio-frequency ablation outcome (Paredes et al., 2020; Pollnow et al., 2020), and low-energy defibrillation techniques (Li et al., 2011; Ji et al., 2017; Uzelac and Fenton, 2020). However, for a complete understanding of arrhythmic mechanisms, simultaneous measurements of *V*_m_ and [Ca^2+^]_*i*_ are needed. Simultaneous measurement of *V*_m_-[Ca^+2^]_*i*_ reveals spatial dispersion of AP repolarization and [Ca^+2^]_*i*_ transients (CaT) (Uzelac et al., 2017) which is one of the mechanisms leading to ventricular arrhythmias (Pastore et al., 1999; Watanabe et al., 2001; Gizzi et al., 2013).

Historically, the first dual optical mapping systems were designed in 2000 by Choi and Salama (2000) with RH-237 V_*m*_ and Rhod-2 AM [Ca^+2^]_*i*_ dyes, and by Laurita and Singal (2001) with Di-4-ANEPPS V_*m*_ and Indo-1 AM [Ca^+2^]_*i*_ dyes. These systems used overlapping excitation bands for the two dyes with separate emission bands split and sent to the two photodetectors, one to measure V_*m*_ and one for [Ca^+2^]_*i*_ with a spatial resolution of 16 × 16 pixels. Since then, other dual systems with higher resolution (Holcomb et al., 2009) have been developed, including those designed with a single camera for monolayers (Scull et al., 2012) and whole hearts (Lee et al., 2011, 2012a,b,c; Herron et al., 2012). The advantages of a single detector based systems include: (i) Significantly less expensive as they do not require multiple detectors/cameras; (ii) There is no need for spatial alignment of the detectors to have the same field of view, increasing complexity of setups, and possible decrease of the field of view (Holcomb et al., 2009); (iii) No need to use a dichroic mirror to separate the V_*m*_ and [Ca^+2^]_*i*_ fluorescence signals for each sensor, decreasing light intensity, that is signal to noise ratio (SNR).

While optical mapping recordings have been considered technically challenging and expensive, advances in recent years have made the necessary equipment affordable enough (Marina-Breysse et al., 2021) to become a standard technique in many labs. Different methods have been proposed to achieve dual *V*_m_ and [Ca^2+^] optical mapping measurement. Two-camera setups require complex spatial alignment of the cameras and optical elements to split the two fluorescent signals, with only one signal reaching each camera (Holcomb et al., 2009). The complexity of the two-camera setups can be avoided by a single camera for dual *V*_m_ and [Ca^2+^]_*i*_ signal measurements. The fundamental principle behind single-camera-based methods is to separate the *V*_m_ and [Ca^2+^] fluorescence signals based on differences in excitation and/or emission spectra of the two dyes used. Existing methods (Lee et al., 2011, 2012a,c) are limited to the particular choice of fluorescent dyes, significantly limiting their application. Additionally, there is no established methodology for designing and implementing a single-camera-based dual *V*_m_ and [Ca^2+^]_*i*_ measurement technique which provides zero-cross talk of the two fluorescence signals. The excitation light band for a *V*_m_ dye preferably would not excite a [Ca^2+^]_*i*_ dye and vice versa. However, true spectral separation is not possible (with currently available dyes), but illuminating at a wavelength where one or the other dye is not sensitive to the parameter of interest is sufficient. This is essential as the complex bidirectional coupling between *V*_m_ and [Ca^2+^]_*i*_ (Shiferaw and Karma, 2006) is important when investigating under which HF conditions (Gorski et al., 2015) *V*_m_ or [Ca^2+^]_*i*_ are responsible for triggering an arrhythmia.

This study presents a theoretical framework to analyze and select the optimal dyes/filters combination to achieve zero-cross talk for dual *V*_m_ - [Ca^2+^] optical mapping applications for simultaneous measurement of *V*_m_ and [Ca^2+^]_*i*_ signals, using a single sensor. The methodology is based on alternating excitation bands for each fluorescent dye in sync with the camera, recording each signal in alternating frames in order to overcome the challenges of mutual cross-talk of the two signals. Excitation at wavelengths we term semasbestic results in no change of fluorescence of a voltage-sensitive dye as *V*_m_ changes, which is suitable to excite a [Ca^2+^]_*i*_ dye with no cross-talk. We demonstrate the existence of a family of these excitation wavelengths and the advantage of this methodology experimentally with optical mapping measurements performed in six different animal species, while also showing how previous methods exhibit signal cross-talk. Furthermore, optical-mapping methods using *V*_m_ and [Ca^+2^]_*i*_ dyes have a broad range of applications, suitable for research of cardiomyocyte cultures (Fast and Ideker, 2000), studying drug effects on heart electrophysiology (Bedut et al., 2016; Streit and Kleinlogel, 2018; Gunawan et al., 2021; Uzelac et al., 2021), gene therapies on *V*_m_ and [Ca^2+^]_*i*_ dynamics in cardiac tissue and in other organs such as the brain (Baker et al., 2005; Ma et al., 2016; Turrini et al., 2017) as well as in other biological systems driven by *V*_m_ and [Ca^2+^]_*i*_ dynamics like in C-Elegans (Venkatachalam et al., 2016).

## 2. Materials and Methods

Previously published methods of simultaneous measurement of *V*_m_-[Ca^2+^]_*i*_ with a single sensor (Lee et al., 2011, 2012b,c), have incorrectly used the term *isosbestic* point of a *V*_m_ dye as a suitable excitation wavelength (λ_Exc_) for [Ca^2+^]_*i*_ dye, expecting no change in measured *V*_m_ fluorescence. While *isosbestic* points (Ahmed and Connor, 1979; Shynkar et al., 2004; Tai et al., 2004; Nič et al., 2009; Bachtel et al., 2011; Uzelac et al., 2019) are defined as the excitation wavelengths at which the total absorbance of a fluorescent dye does not change in response to a change of *V*_m_ (Figure 1A; Supplementary Figure 1), it was instead applied as the excitation wavelength at which there is no measurable change in *V*_m_ fluorescence signal, expressed as Δ*F*/*F* = (*F*−*F*_0_)/*F*_0_≈0, where *F*_0_ is the fluorescence intensity when the cell's membrane is at the resting membrane potential, and *F* = *F*(*V*_m_) is the intensity at any other cell membrane potential.

Isosbestic point is defined as the excitation wavelength at which the absorption spectra for polarized cell membrane and the shifted absorption spectra for maximally depolarized membrane, intersect each other (Bachtel et al., 2011) (Figure 1A). Therefore, when illuminating at an isosbestic point, the absorption coefficient changes minimally (can be approximated to zero), and the integral over the entire emission spectra remains the same. This implies Δ*F*/*F* = 0, only if the LPF placed on the camera side passes the entire emission spectra. In practice, this is not possible as cameras have limited dynamic rang, and in common use the LPF on the camera side blocks part of the emission spectra to increase the absolute Δ*F*/*F* value (Figure 1A).

In this study, we show that *isosbestic* points, which are an intrinsic property of fluorescent dyes and as such do not depend on the optical filters transmission properties and sensor spectral response used in many optical mapping setups, are not necessarily the correct wavelengths to use to prevent cross-talk and in fact can give large *V*_m_ signal (see top panel *V*_m_ signal in Figure 1D obtained with the same optical filter values and the same camera used in Lee et al. (2012b) what authors mistakenly considered to be an isosbestic point excitation). In this study, we establish a methodology to design bi-modal optical-mapping systems with the selection of the correct optical filters, given a choice of *V*_m_ and [Ca^2+^]_*i*_ dyes. We demonstrate that for a given electrochromic *V*_m_ dye, a continuous range of excitation wavelengths exist that result in Δ*F*/*F* = 0 (no fractional change of fluorescence) dependable on a given a LPF cut-on wavelength (λ_LPF_) and the spectral response of the camera used. We have termed such a family of wavelengths as *semasbestic* (self-extinguishable) wavelengths, which are a function of the λ_LPF_ for a particular *V*_m_ dye. *Thus*, *V*_m_ *and [Ca*^2+^*]*_*i*_ *dyes can be excited with different wavelengths in alternating frames, the* *V*_m_ *with a wavelength outside of a [Ca*^2+^*]*_*i*_ *dye's absorption spectrum and the [Ca*^2+^*]*_*i*_ *dye within the absorption spectra of the* *V*_m_ *dye, but using a semasbestic wavelength for the* *V*_m_ *dye, to achieve zero cross-talk for both signals*.

The absorption and emission spectra of electrochromic *V*_m_ dyes bound to a cardiac cell membrane and shift a few nanometers toward blue as the membrane depolarizes (Loew, 1982) (Figure 1A). The *isosbestic* point, is the intersection of the absorption curves for the polarized (−80 mV) and fully depolarized (approximately +20 mV) membrane (Figure 1A), typically occurring near the peak of the absorption spectrum (Ahmed and Connor, 1979; Bachtel et al., 2011; Uzelac et al., 2019). Excitation at the *isosbestic* point results in a leftward shift of the emission curve without changing in amplitude. Therefore, the only case in which no change in *V*_m_ signal can be measured (Δ*F*/*F* = 0), occurs when using an LPF on the camera side that passes the entire emission spectra for both (polarized/depolarized cell membrane) curves (Figure 1A).

Excitation with wavelengths shorter than the *isosbestic* (Figure 1A) increases the absorbed light, resulting in an increased amplitude of the shifted emission spectrum. For the emission spectrum range, approximately left of the emission peak, Δ*F*/*F* is positive, and for the range right of the peak, Δ*F*/*F* is negative. With LPF on the camera side, the actual measured fluorescence signal represents integrated emission spectra from the filter λ_LPF_, and the overall sign of Δ*F*/*F* can be positive or negative depending on λ_LPF_. A *semasbestic* wavelength will be then the excitation wavelength such that integrated intensity over the emission spectra starting from λ_LPF_ results in Δ*F*/*F* = 0. Therefore, *Semasbestic* wavelengths depend on the λ_Exc_ and the λ_LPF_ used, resulting in continuous line of Δ*F*/*F* = 0 values (Figure 1B).

To demonstrate the existence and usefulness of *semasbestic* wavelengths, we used the near infra-red electrochromic *V*_m_ dye JPW-6003 (Supplementary Figure 1) in isolated Langendorff perfused hearts of six animal species, fish (*N* = 2), Guinea pigs (*N* = 2), rabbits (*N* = 12), cats (*N* = 6), pigs (*N* = 7), and sheep (*N* = 2), totaling 31 experiments, in addition to monolayers cultured from neonatal rat hearts. Details of experimental materials and methods, including heart excision and preparation, cell culture monolayers preparation, excitation light sources, emission optical filters characterization, absorption and emission spectra of the JPW-6003 V_*m*_ dye, as well as methods used to obtained Δ*F*/*F* = 0 *semasbestic* points experimentally and their statistical analysis, are provided in the Supplementary Materials.

## 3. Results

Δ*F*/*F* values were measured in isolated heart experiments stained with *V*_m_ dye JPW-6003, and excited with a series of different excitation light bands using 10 nm wide bandpass (BP) excitation filters of nominal center wavelengths ranging from 500 to 671 nm. The filters were coupled to either green or red LED collimated light (Figure 1C), and for four different LPFs used on the camera side (Supplementary Figure 2). The excitation light band of each BP filter was modeled with a single effective excitation wavelength (Supplementary Figure 3).

For each LPF-BP filter combination (out of the 60 possible), optical mapping recordings of *V*_m_ were obtained for a duration of 2–10 min (depending on the amplitude of Δ*F*/*F* signal), acquiring a sequence of images with the *V*_m_ across the tissue, at 500 FPS at a resolution of 128 × 128 pixels. For signals of low Δ*F*/*F* values close to the *semasbestic* wavelengths, where signal to noise ratio (SNR) decreases (Figure 1D), the longer recordings (10 min) were used to perform action potential (AP) stacking (ensemble-averaging) (Uzelac and Fenton, 2015) in post-processing. The stacking procedure significantly improved SNR to precisely obtain the small Δ*F*_max_/*F* magnitude values (Figure 1D; Supplementary Figure 4).

**Figure 2:**
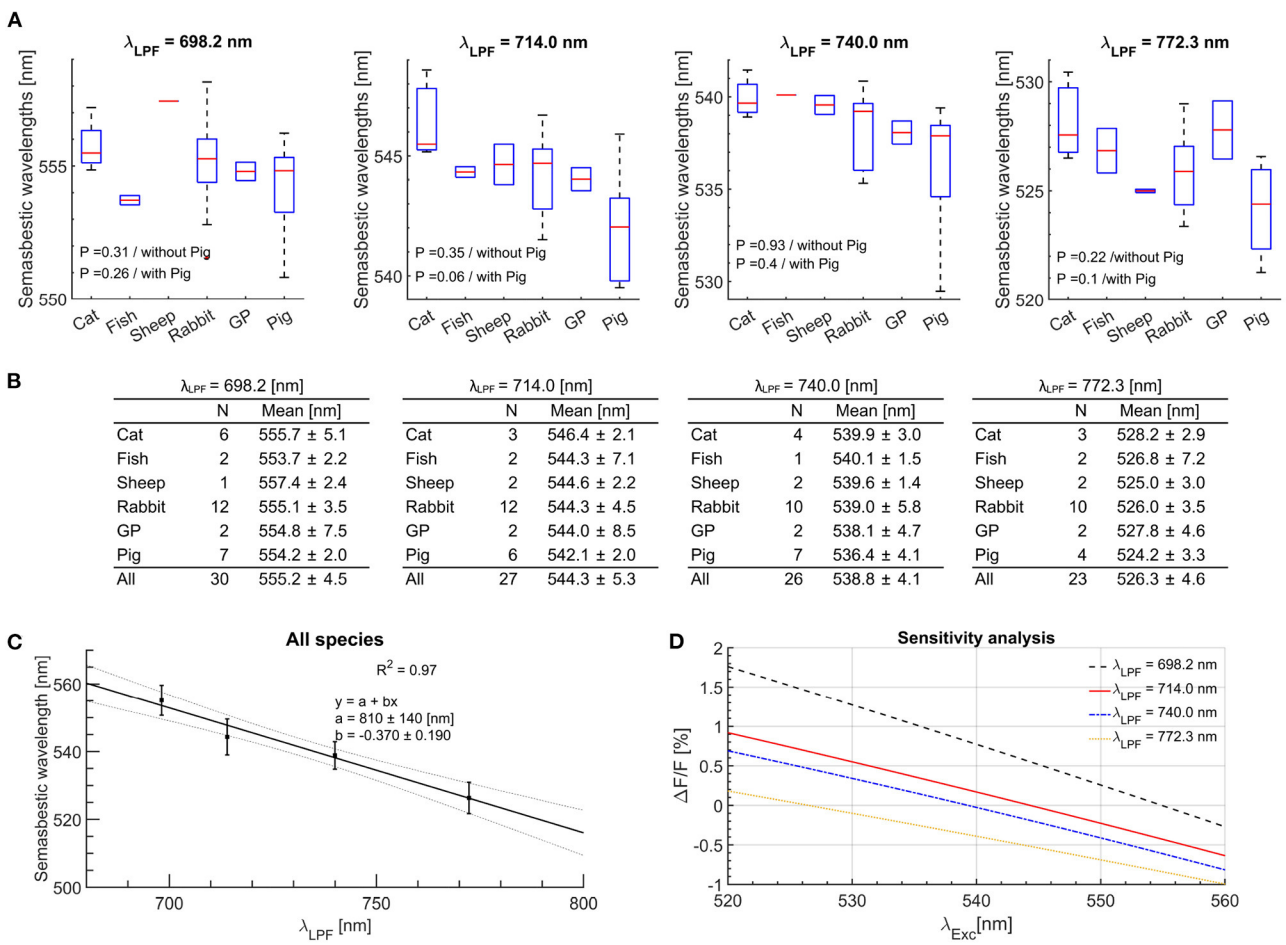
The *semasbestic* wavelengths across all experiments for JPW-6003 *V*_m_ dye. **(A)** Box plots are a statistical representation of *semasbestic* wavelength averaged for each species. The ends of each box are the upper and lower quartiles, with the median marked as a horizontal line inside the box. The whiskers lines represent the upper and lower extremity. The P-values represent results of one-way ANOVA analysis with and without the *isosbestic* points from isolated pig hearts experiments. **(B)** The mean values and uncertainties of experimentally obtained *semasbestic* points across all species for different LFPs used on the camera. **(C)** A linear curve fit relating the range of *semasbestic* wavelengths corresponding to different λ_LPF_. **(D)** Sensitivity analysis near the *isosbestic* wavelength for different LPFs. Experimentally obtained Δ*F*/*F* magnitude values were averaged across all species for the same LPF-BP filter pairs. Excitation wavelengths for up to 10 nm off the *semasbestic* wavelength result in less than 0.5% in the fractional change of the *V*_m_ signal.

For each isolated experiment and each LPF, *semasbestic* wavelengths were obtained as zero values of the fit curves of Δ*F*/*F* magnitudes obtained for different excitation BP filters. The range of semasbestic wavelength hypothesized theoretically matches well with those obtained experimentally (Figures 1E,F). Among obtained *semasbestic* wavelengths from the different animal species, no statistically significant difference was observed, with the largest variation found only in pig hearts (Figure 2A). We attribute this difference to the surface fatty tissue layer (that other animals did not have) attenuating non-linearly the emitted fluorescence spectra (van Veen et al., 2004), which resulted in a small leftward shift of the *semasbestic* wavelengths. One-way ANOVA tests were performed with and without the *semasbestic* wavelengths obtained in pig hearts, and found that even when including the *semasbestic* wavelengths of the pig hearts, *P*-values did not reach statistical significance (*P* < 0.05 was considered statistically significant). Therefore, *semasbestic* wavelengths (Figure 2B) appear to be independent of animal species, and a curve can be fitted to relate *semasbestic* wavelengths as a function of λ_LPF_ for *V*_m_ JPW-6003 dye, (Figure 2C).

Any *semasbestic* wavelength is suitable to excite a ${\left[Ca^{2+}\right]}_{i}$ dye. However, in practice using off-the-shelf filters it is expected some mismatch with the filter effective excitation wavelength from the ideal *semasbestic* wavelength. Any mismatch will result in a cross-talk, the presence of the *V*_m_ signal while measuring the ${\left[Ca^{2+}\right]}_{i}$ fluorescence signal. However, for the excitation wavelengths of up to 10 nm from the ideally suitable *semasbestic* wavelength, the fractional change of *V*_m_ fluorescence is less than 0.5% (Figure 2D). Staining a heart in addition with a ${\left[Ca^{2+}\right]}_{i}$ dye and assuming the same baseline fluorescence levels of the two dyes, the fractional change of 0.5% will results in ~0.25% fractional change of both fluorescence signals combined, attributed to *V*_m_−*Ca* cross-talk. In practice for *V*_m_−*Ca* single-camera measurements, a relative fluorescence change measured in *Ca* fluorescence channel, of for example 5% means that 0.25% of the measured fluorescence change is attributed to the unwanted *V*_m_ signal.

**Figure 3:**
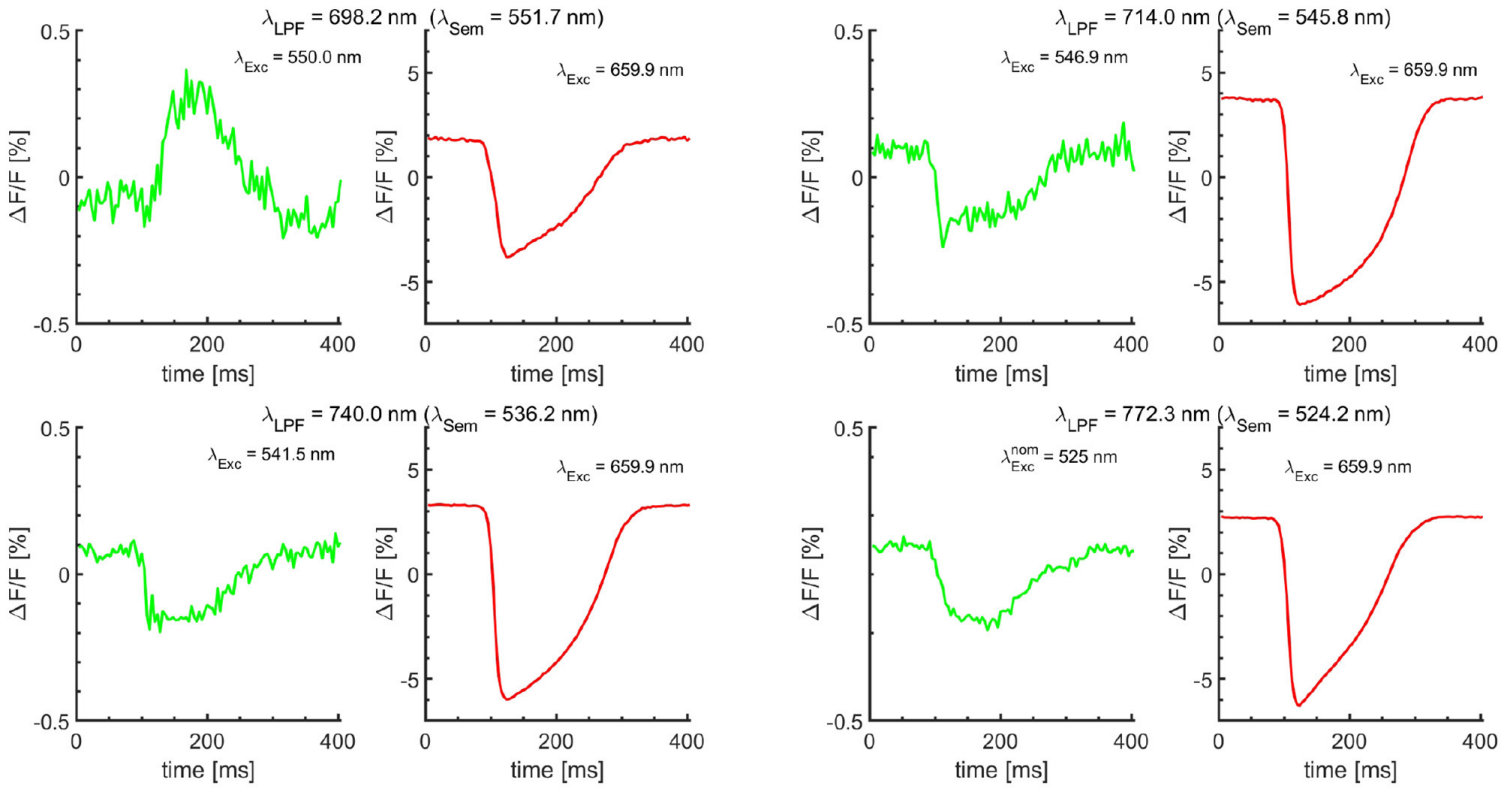
Single-camera dual *V*_m_ measurements. The *V*_m_ dye was excited with alternating excitation bands, using a 660/10 excitation BP filter in even frames. Odd frames were acquired using off-the-shelf excitation BP filters selected to closely match the *semasbestic* points (λ_Sem_) corresponding to the λ_LPF_ of the four LPFs. λ_*Exc*_ wavelengths are effective excitation wavelengths of the BP filters. Their nominal center wavelengths are listed in Supplementary Figure 3. $\lambda_{\text{Exc}}^{nom}$ = 525 nm is the nominal center wavelength of 20 nm wide (OD6) BP filter (Semrock). Shown optical action potential signals are obtained using the stacking procedure from a single pixel, averaging at least 400 periods without any filtering. The stacking resolves small *V*_m_ signal changes buried under the noise level, which could be interpreted as no change in *V*_m_ signal in the odd frames otherwise. The cross-talk, the presence of *V*_m_ signal due to the differences between the filters effective excitation wavelength and corresponding *semasbestic* points is less than 0.25% for most LPF. The sensitivity to the difference is the lowest for λ_LPF_ = 740.0 nm, resulting in Δ*F/F* amplitude of less than 0.25% using excitation wavelength of more than 5 nm different from the ideal semasbestic point.

To further demonstrate the proof of concept for *V*_*m*_−*Ca* cross-talk minimization for each of the four LPF filters, *V*_m_ measurements with JPW-6003 dye only were performed using alternating excitation bands in sync with the camera frame rate (500 Hz at a resolution of 128 × 128 pixels). The sequence of images was recorded with the odd frames corresponding to ideally no change in *V*_m_ fluorescence (DFF = 0) and even frames corresponding to the change in *V*_m_ signal fluorescence (Figure 3). For odd frames, the off-the-shelf BP excitation filters were used chosen to match as close as possible the ideal *semasbestic* point corresponding to each of the four different LPFs, and coupled with 525 centered green LED. For even frames, the BP excitation filter of the effective λ_*Exc*_ = 660.0 nm wavelength was used. For each of the four validation tests with different LPF, the amount of *V*_*m*_−*Ca* cross-talk is around 0.25% except for λ_*LPF*_ = 698.2, which is around 0.4%. The cross-talk is represented as a fractional change in the *V*_m_ dye fluorescence. The optical action potential traces corresponding to the same image pixel for even and odd image sequences for direct comparison. As off-the-shelf BP filters were used, the amount of cross-talk depends on the difference between the BP filter effective wavelength from the semasbestic point. Based on the measurement (Figure 3), sensitivity is minimal for $\lambda_{\text{LPF}}^{nom}$ = 740.0 nm, using a BP filter of effective excitation wavelength of 5 nm off the *semasbestic* point and resulting in Δ*F*/*F* less than 0.25%.

## 4. Discussion

The range of different *semasbestic* wavelengths of a given *V*_m_ dye provides flexibility in the design of an optical mapping system for simultaneous single-camera *V*_m_ - *Ca* recordings, as it is not limited to a single choice of excitation and emission filters, and *V*_m_ and *Ca* fluorescence dyes. The design parameters include the optimal excitation wavelength for a *V*_m_ dye (that maximizes Δ*F*/*F*), choice of [Ca^2+^]_*i*_ dye and its excitation wavelength (*semasbestic* point), the dual-band pass optical filter on the camera side, and suitable LEDs coupled with excitation filters. Availability of LEDs, optical excitation filters, and the dual-band pass filter of desired spectral properties based on the theoretical design are additional constraints that need to be considered. To begin with the parameters optimization, the first step is to choose a *V*_m_ dye.

### 4.1. *V*_m_ Dye Selection

Selection of the *V*_m_ dye determines the range of *semasbestic* wavelengths. For optical mapping method optimization with a chosen *V*_m_ dye, the first step is to determine the *V*_m_ dye emission spectra to optimize Δ*F*_max_/*F*. For a chosen JPW-6003 dye its absorption and emission spectra are shown in Supplementary Figure 1. Excitation of the *V*_m_ dye with longer wavelengths than the *isosbestic* point and using longer λ_LPF_ on the camera side, increases Δ*F*_max_/*F* magnitude (Figures 1B–F; Supplementary Figure 5). However, the SNR decreases for longer λ_LPF_ due to less fluorescence light reaching the detector. A common practice is to choose λ_LPF_ between the *V*_m_ dye emission peak and 50% of the peak, the range from 708 to 775 nm for JPW-6003 dye (Supplementary Figure 1). Considering suitable and currently available deep red high-power LEDs, the optimal choice is to use peak emission LEDs around 660 nm.

### 4.2. [Ca^2+^]_*i*_ Dye Selection

Selection of the suitable [Ca^2+^]_*i*_ dye is constrained with the range of the *semasbestic* wavelengths corresponding to the selected range of λ_LPF_ values, from 708 to 775 nm. From the equation shown in Figure 2C, the corresponding range of *semasbestic* wavelengths range from 523 to 548 nm, suitable to excite [Ca^2+^]_*i*_ dyes such as Cal-520, Rhod-2, and Rhod-4. In this study, we choose Rhod-2 dye with a peak absorption at 552 nm. However, commercially available LEDs offer peak emission around 525 nm, decreasing emission intensity toward the Rhod-2 absorption peak, making excitation at 540 nm a suitable trade-off. Another important aspect to consider is to avoid overlap of the [Ca^2+^]_*i*_ dye absorption spectra with the chosen JPW-6003 *V*_m_ dye excitation wavelength of 660 nm. Rhod-2 [Ca^2+^]_*i*_ dye absorption spectrum effectively reaches zero above 625 nm, making Rhod-2 dye a suitable choice for [Ca^2+^]_*i*_ measurement.

### 4.3. Dual-Band Pass Filter

A dual-bandpass optical filter is required on the camera to pass the emitted fluorescence from both dyes. With the chosen 540 nm *semasbestic* wavelength, the *V*_m_ dye band is determined from the λ_Exc_ vs. λ_LPF_ curve (Figure 2C) to start at ~730 nm. The first filter band corresponding to the Rhod-2 emission spectra can be from 560 and up to 610 nm. The lower band limit is imposed by excitation BP filter centered at 540 nm so that its transition band effectively reaches zero (<10^−4^) at 560 nm. The upper limit is determined to avoid overlap with the *V*_m_ emission spectra to minimize cross-talk, the presence of *V*_m_ signal in the 560–610 nm filter band when the Rhod-2 dye is excited (Supplementary Figures 1, 6).

### 4.4. Excitation Filters

The LED bell-shaped emission curve results in non-uniform excitation spectra passing through the BP excitation filter. For example, the light intensity passed through the 10 nm wide 540 nm centered BP filter is higher for the wavelength range left of the 540 nm than for the range right of the 540 nm, resulting in different effective (mean) excitation from the nominal 540 nm center wavelength. Since this effective excitation wavelength has to match the *semasbestic* wavelength, any mismatch will result in a non-zero Δ*F*/*F* value. However, the introduced error is small, and the *V*_m_ signal change is less than 0.5% using an excitation wavelength in the ±10 nm range around the *semasbestic* wavelength. The fractional changes were measured only with the *V*_m_ dye. Adding the *Ca* dye, the amount of cross-talk will be even lower, as the fractional change is measured in respect to the summed baseline fluorescence of both dyes (Figure 2D). For the *V*_m_ dye excitation, the width and spectral non-uniformity of the passed light are not critical. With a 660 nm centered LED, BP filters with a 660 nm center wavelength of up to 50 nm BP width can be used. The limits are imposed to avoid excitation of the Rhod-2 dye, and a practical limit is due to the LED's bell-shaped emission curve.

### 4.5. Practical Design With Applications

The optical mapping setup with optimized parameters may come at a high cost requiring the manufacturing of custom filters. However, flexibility in the choice of the *semasbestic* wavelengths provides leverage in the choice of design parameters while still minimizing the cross-talk for single-camera based simultaneous *V*_m_ - *Ca* measurements to use off the shelf filters. The dual BP filter is the most important for the system design as it determines the *semasbestic* wavelength. Additionally, any mismatch between the off-the-shelf BP filter effective excitation wavelength and the *semasbestic* wavelength will result in *V*_m_ to *Ca* cross-talk. However, as outlined above, the amount of cross-talk is less than 0.25% when the difference between the BP filter effective excitation wavelength and the *semasbestic* point is less than 5 nm (Figures 2D, 3).

For practical realization, we choose an off-the-shelf dual BP filter, of optical density (OD) 6 (Chroma), with the first passband of 560–610 nm, and the second LPF band of the nominal 700 nm wavelength (effective 698.2 nm) (Supplementary Figure 7). Based on the equation (Figure 2C), the corresponding *semasbestic* wavelength for JPW-6003 *V*_m_ dye is 551.7 nm. The closest off-the-shelf 10 nm wide BP filter of 550 nm (nominal wavelength) center wavelength (Edmund Optics) was chosen to be used for Rhod-2 *Ca* dye excitation (Supplementary Figure 3). An additional OD6 LPF of nominal λ_LPF_ = 575 nm (Chroma) was placed over the dual BP filter to avoid overlap between the 550/10 BP excitation filter and the 560–610 nm passband of the dual-band pass filter. This design narrows the Rhod-2 *Ca* emission spectra range to the 575–610 nm range resulting in reduced *Ca* fluorescence intensity reaching the camera sensor. However, this was a necessary trade-off using off-the-shelf optical filter filters. For the *V*_m_ dye excitation, a 10 nm wide OD4 BP filter of 660 nm nominal center wavelength was used (Edmund Optics). A green LED with 525 nm peak (Luminous Devices) coupled with 550/10 nm BP filter for Rhod-2 *Ca* dye excitation was used, and a red 660 nm peak LED (LEDEngin) coupled with the 660/10 nm BP for *V*_m_ dye excitation (Supplementary Figure 8).

**Figure 4:**
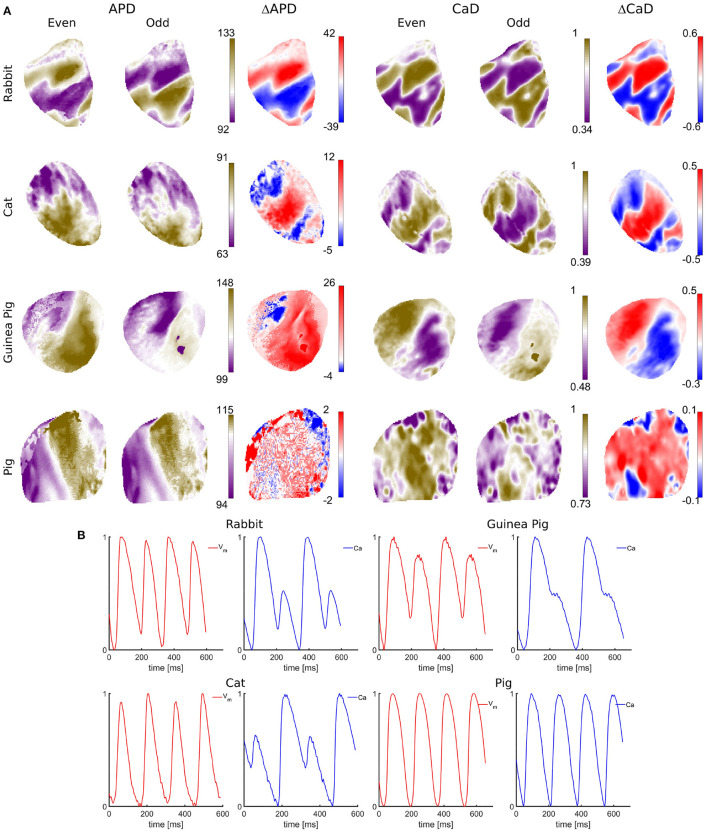
Single-camera dual *V*_*m*_−*Ca* measurements in isolated hearts of different species. **(A)** Arrhythmic effects under dynamic pacing, shown as spatial dispersion of APD and CaD across tissue for different species for even and odd beats. The spatial dispersion indicates an increased susceptibility to arrhythmia. APD values are obtained from 50% signal rise in amplitude till 50% AP repolarization. Numbers to the right indicate 3rd and 97th percentile APD values expressed in milliseconds. CaD values are obtained as the integral from 50% rise in amplitude till 50% decrease. Blue-red patterns show variations in APD and CaD (ΔAPD, ΔCaD) between even and odd beats (discordant alternans), showing regions alternating out of phase and separated with the nodal lines (white lines). Spatially discordant alternans are the counterpart of T-wave ECG alternans, a well-known marker for arrhythmia susceptibility. Although all species do develop arrhythmia under dynamic pacing, the electrophysiology across species is very different. Spatially discordant alternans are the most pronounced in rabbits, while pig hearts show very little APD and CaD dispersion, with insignificant differences between even and odd beats. **(B)** *V*_*m*_ and *Ca* representative AP signals showing temporal alternans for different species.

**Figure 5:**
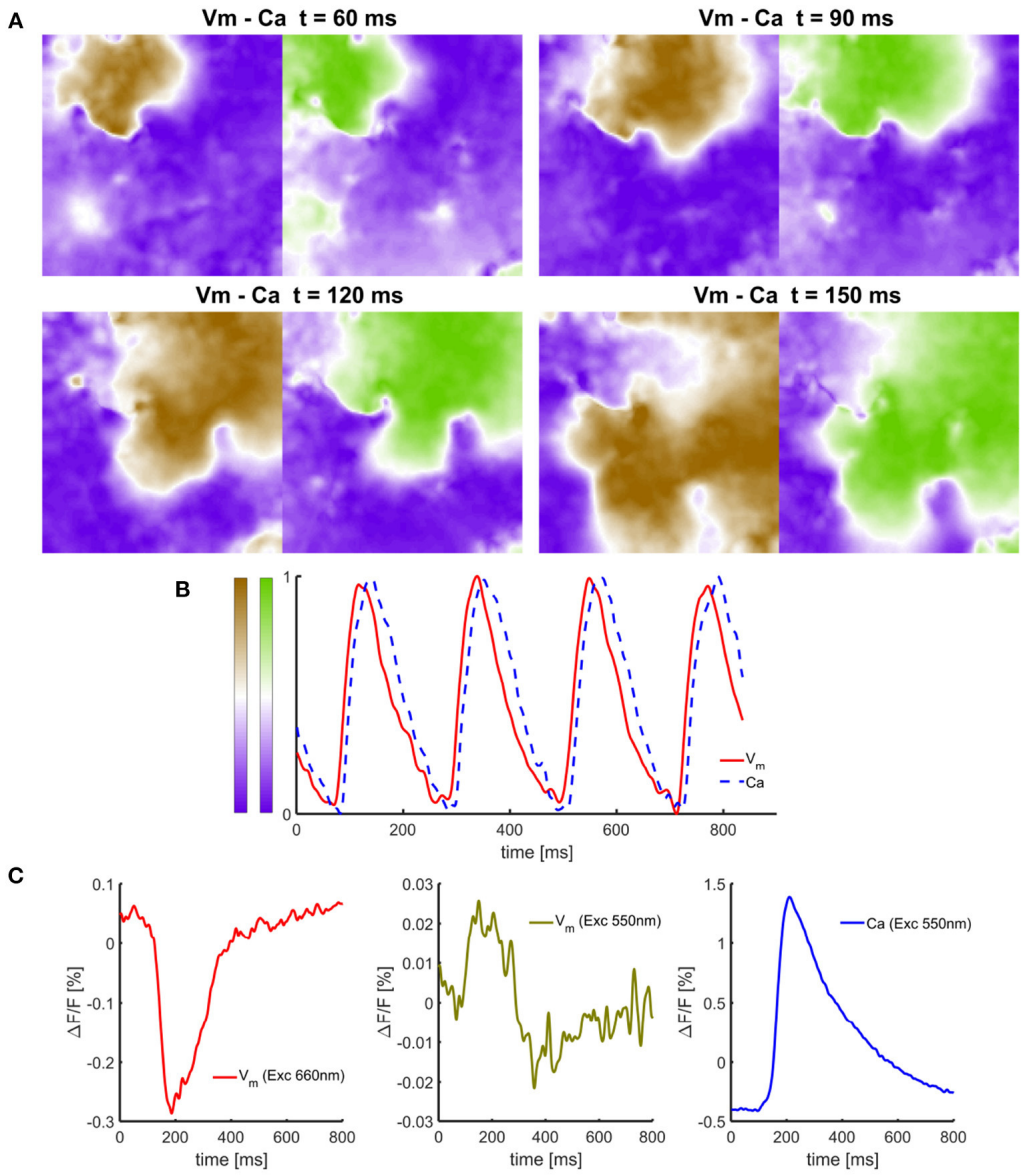
Simultaneous single-camera-based measurements of *V*_m_ and Ca signals in isolated cell culture monolayers of neonatal rats. **(A)** Time-snapshots of *V*_m_ - *Ca* dynamic at different time points illustrating the presence of anchored spiral wave and spatial loss of correlation between propagating *V*_m_ wavefront from *Ca* dynamics. **(B)** Normalized *V*_m_ and *Ca* signals obtained from processed raw recordings showing *Ca* signal lagging in time. **(C)** Representative unfiltered *V*_m_ and *Ca* signal traces with no filtering expressed as a relative change. In cell culture monolayers, *Ca* signals have significantly higher SNR than *V*_m_ signals originating only from the dye bound to the cell membrane. The amount of cross-talk and the presence of *V*_m_ signal in *Ca* signal with excitation at near the semasbestic point (Exc550) results is a small yet negligible cross-talk compared to the amplitudes of the signals.

Quantification of spatiotemporal discordant alternans of *V*_m_-[Ca^2+^]_*i*_ in cardiac tissue. The optical mapping system with the design parameters described above was used to measure *V*_m_ - [Ca^2+^]_*i*_ signals with a single-camera in isolated hearts of Rabbit, Cat, Guinea Pig, Pig (Figure 4), and monolayer cell culture of neonatal rats (Figure 5). A restitution protocol was performed for each species, where *V*_m_ - *Ca* signals were recorded at decreasing pacing cycle lengths (PCLs) until a conduction block or ventricular fibrillation (VF) occurs. At shorter PCLs, instabilities in *V*_m_ or [*Ca*_i_] at the cellular level lead to the development of beat to beat alternans in action potential duration (APD) (Pastore et al., 1999), and intracellular calcium duration (CaD) (Uzelac et al., 2017). Through tissue-level coupling, these instabilities lead to complex and irregular spatial dispersion in AP repolarization and in CaD (Figure 4), forming dangerous spatially discordant alternans. Spatially discordant alternans are equivalent to T-wave alternans in clinical ECGs (Pastore et al., 1999; Uzelac et al., 2021), the clinical marker for arrhythmia susceptibility and sudden cardiac death (Walker and Rosenbaum, 2003; Verrier et al., 2009). Among species, the differences in ionic membrane channel densities such as potassium repolarization channels and lack of specific ionic channels create differences in AP morphology. In addition, through *V*_m_ - *Ca* bidirectional coupling, other differences across species exist in terms of handling the intracellular *Ca* cycling (Figure 4). For rabbit, cat, and guinea pig heart, alternans in *Ca* seem to drive *V*_m_ alternans leading to spatial dispersion of AP repolarization. In contrast, no alternans are observed in *V*_m_ nor *Ca* signals for pig hearts, yet pig hearts develop VF at faster PCLs in the restitution protocols. Understanding these differences across species could lead to a better understanding of different arrhythmia mechanisms modalities and relate them to human heart physiology to more complete understanding arrhythmia in human hearts, devise novel treatments, and help with the global epidemic of HF.

## 5. Conclusions

This study presents a methodology to achieve zero cross-talk (smaller than SNR after stacking) in a single-camera bi-modal optical mapping design based on *semasbestic* wavelengths. The presented guidelines show how to optimize *semasbestic* wavelengths to achieve zero cross-talk among *V*_m_ and *Ca* signals, while optimizing signals amplitudes as well. With off-the-shelf bandpass excitation filters of slightly different effective excitation wavelengths than corresponding *semasbestic* wavelengths (Figure 3) we achieved near zero-cross talk. For a true zero cross-talk, custom manufactured bandpass filters matching the *semasbestic* point would be needed, and one would need to take into account the LED excitation light spectral profile within the filter passband. The absorption spectra of a given electrochromic *V*_m_ dye is spectrally shifted when the dye is bounded within a cell membrane due to a strong interaction between the dye's molecule electric dipole momentum and the cell membrane electric field (Matson et al., 2012). Therefore, contemporary spectroscopy methods to obtain the dye absorption and emission spectra, dissolving the dye in a solvent such as ethanol and using a spectrometer, would result in different *semasbestic* wavelengths than when performing equivalent measurements on isolated hearts stained with the dye. In this study we conclude that obtained *semasbestic* wavelengths for a given electrochromic *V*_m_ dye, are dependent only on the excitation wavelength λ_Exc_ and corresponding λ_LPF_ of the dye emission band. As validated on isolated hearts of six different species, the *semasbestic* wavelengths seems to be independent of the species (as validated on six different species and cell culture monolayers).

In the published literature of simultaneous measurement of *V*_m_-[Ca^2+^]_*i*_ with a single sensor (Lee et al., 2011, 2012b,c), authors used the same EMCCD camera model and the dual-bandpass filter of 700 nm LPF cut-on wavelength for the JPW-6003 *V*_m_ fluorescence showing no visible *V*_m_ signal when excited with 540 nm centered band, subsequently used for Rhod-2 Ca dye excitation. However, our findings are different. As shown in Figure 1D, excitation at 540 nm with LPF of 700 nm nominal cut-on wavelength resulted in apparent presence of *V*_m_ signal. Therefore, exiting Rhod-2 dye with 540 nm centered bandpass filter and using the dual-band pass filter of 700 nm LPF band for *V*_m_ fluorescence results in a cross-talk which can be further minimized with the presented methodology for cross-talk elimination.

While the presented methodology is demonstrated with the *semasbestic* wavelengths of JPW-6003 *V*_m_ dye and using Rhod-2 [Ca^2+^]_*i*_ dye, the described methodology does not depend on the choice of the dyes. It applies to any other electrochromic *V*_m_ resulting in a different set of *semasbestic* wavelengths. Therefore, depending on the user's need, we provide a range of *semasbestic* excitation wavelengths that can be used with JPW-6003 *V*_m_ sensitive dye, and a methodology to obtain the same for other *V*_m_ sensitive dyes. We followed the common practice in optical mapping to use an LPF to record *V*_m_ signal fluorescence. Excitation on the positive-slope side of the excitation spectrum induced an increase in fluorescence during an action potential, which is balanced by recording from the negative-slope side of the emission spectrum. Other classes of *semasbestic* points may exist with different designs of the emission filter. For example, it is also possible to create a *semasbestic* wavelength along the negative-slope side of the excitation spectrum and balance this by recording from a band on the positive-slope side of the emission spectrum. However, the close separation between the dye absorption and emission spectra (Supplementary Figure 1) may preclude such an approach for JPW-6003 dye, while it may be feasible for other *V*_m_ dyes.

Optical mapping methods using fluorescent dyes are well-established and very important to measure signals from cells and tissue. The use of optical mapping systems is rapidly growing. Nowadays, it is becoming a common tool in many research labs as the equipment is no longer prohibitively expensive (Lee et al., 2017) with novel advances in CMOS sensor technology. The increasing number of research groups are using optical mapping to investigate *V*_m_-[Ca^+2^]_*i*_ on biological tissues, and optical mapping methods are even becoming accessible in university classrooms. Simultaneous measurement of *V*_m_ and [Ca^2+^]_*i*_ enables many studies in the area of electrophysiology related to the *V*_m_ and [Ca^2+^]_*i*_ dynamic and their bidirectional coupling. For example, cardiac arrhythmia can be caused by AP spatial dispersion driven with *Ca* alternans at the cellular level (Uzelac et al., 2017) (Supplementary Videos 1, 2). Dual *V*_m_ and [Ca^2+^]_*i*_ measurements are necessary to understand the underlying mechanism leading to arrhythmia (Groenendaal et al., 2014), and optical mapping studies can be performed even in combination with contractions (Christoph et al., 2018) to study the mechanical stretching effects on cardiac electrophysiology. Time-resolved series of *V*_m_ and [Ca^2+^]_*i*_ signals at high spatiotemporal resolution are generally applicable to other electrophysiology related studies besides HF. For example, many drugs affect the AP repolarization phase blocking the potassium channels and prolonging the APD, the cellular mechanism of long-QT. Optical mapping provides integrative studies at both the cellular and tissue level to understand how drugs affect the ionic channel currents and intracellular *Ca* dynamics and to understand the drug's safety profile and associated antiarrhythmic or proarrhythmic at the tissue level (Uzelac et al., 2020, 2021), which would not be possible to understand, studying the drug's effect at the cellular level only. The methodology developed here can also be used to investigate other biological systems with *V*_m_-[Ca^+2^]_*i*_ driven dynamics such as the brain (Rad et al., 2017) the pancreas (Yang and Berggren, 2006), smooth (Nelson et al., 1990), and skeletal (Flucher and Tuluc, 2017) muscle among others.

## Data Availability Statement

The raw data supporting the conclusions of this article will be made available by the authors upon reasonable request.

## Ethics Statement

The animal study was reviewed and approved by the Office of Research and Integrity Assurance at Georgia Institute of Technology and T3 Labs (Translation Testing and Training Laboratories, Inc.), Institutional Animal Care and Use Committee (IACUC) protocols A18012 and A15002.

## Author Contributions

IU performed the study design, designed and performed the experiments, and analyzed and interpreted the data. CC contributed to data interpretation, theoretical postulations, and calculations. SI provided critical comments to the study. HC and TK contributed with isolated monolayer cell culture preparation and assisted in related experiments. FF contributed to study design and experiments. All authors worked in writing and reviewing the manuscript.

## Funding

This study was supported by grants NIH 1R01HL143450-01, National Science Foundation 1446675, American Heart Association 15POST25700285, NHLBI R01HL143065, NHLBI R01HL147270, NHLBI R01HL157363, 20TPA35260085, and Department of Defense GRANT12901705 (PR191598).

## Conflict of Interest

IU is the owner of Aleksa Tech Inc., a manufacturer of power sources for LED illumination in optical mapping measurements. The remaining authors declare that the research was conducted in the absence of any commercial or financial relationships that could be construed as a potential conflict of interest.

## Publisher's Note

All claims expressed in this article are solely those of the authors and do not necessarily represent those of their affiliated organizations, or those of the publisher, the editors and the reviewers. Any product that may be evaluated in this article, or claim that may be made by its manufacturer, is not guaranteed or endorsed by the publisher.
