# Supplementary material for: Methodology for Cross-Talk Elimination in Simultaneous Voltage and Calcium Optical Mapping Measurements With Semasbestic Wavelengths
Source: Front Physiol. 2022 Feb 11;13:812968. doi: 10.3389/fphys.2022.812968 (PMC8874316; doi:10.3389/fphys.2022.812968)
Supplement: Supplementary file 1 [file Data_Sheet_1.pdf]

frontiers

Supplementary

Correspondence*:
Ilija Uzelac, ilija.uzelac@physics.gatech.edu

HEART EXCISION AND PREPARATION

All experiments conform to the current Guide for Care and Use of Laboratory Animals, published by the National Institutes of Health (NIH Publication No. 85 - 23, revised 1996), and approved by the Office of Research and Integrity Assurance at Georgia Institute of Technology and T3 Labs (Translation Testing and Training Laboratories, Inc.), Institutional animal care and use committee (IACUC) protocols A18012 and A15002 for rabbit, guinea pig, and fish. Cat, sheep, and pig hearts were donated from other research groups at Georgia Tech performing non-heart related studies to minimize animal use in research. Before deep anesthesia, New Zealand White rabbits (2 - 3 kg) were lightly anesthetized with ketamine/xylazine/acepromazine (17/9/0.9 mg/kg). Rabbits, cats, pigs, and sheep, were deeply anesthetized with 3 - 5% isoflurane gas mixed with oxygen for induction and approximately 1 - 3% for deep anesthesia maintenance. Five minutes before euthanasia, heparin was injected intravenously, 300 U/Kg for rabbits and cats, and 100 U/Kg for sheep and pigs. Rabbit and cat hearts were excised via left thoracotomy, under deep anesthesia for rabbits, and after euthanasia with intravenous injection of pentobarbital (120 mg/kg) for cats. Sheep and pig hearts were excised via median sternotomy after euthanasia with KCl (100 mg/kg) given intravenously under deep anesthesia until cardiac arrest. Zebrafish were euthanized by either immersion in tricaine methanesulfonate (MS-222) solution (300 mg/L; 25 to 30 °C) buffered with sodium bicarbonate to a neutral pH or immersion in cold water mixed with ice. Fish hearts were excised after 10 minutes of cessation of opercular movement.

After excision, cat, rabbit, sheep and pig hearts were perfused retrogradely via the aorta for 3 minutes with cold cardioplegic solution (NaCl: 110, KCl: 16, $NaHCO_3$: 10, $MgCl{\cdot}6H_2O$: 16, $CaCl_2$: 1.2, all units in mM), gassed with mixture of 95% O_2 / 5% CO_2. Cardioplegic perfusion was used to induce cardiac stasis, protecting the myocardium while hearts are transferred to our optical mapping lab (within 15 minutes). In the lab, the hearts were immersed in a heated oval chamber kept at 37.0 ± 0.5 °C, and Langendorff-perfused with Tyrode's solution (NaCl: 124, KCl: 4, $NaHCO_3$: 24, $NaH_2PO_4{\cdot}H_2O$: 0.9, $MgCl{\cdot}6H_2O$: 0.7, dextrose: 5.5, $CaCl_2{\cdot}2H_2O$: 2, all units in mM), at regulated temperature of 37.0 ± 0.5 °C and gassed with mixture of 95% O_2 / 5% CO_2. Rabbit and cat hearts were perfused retrogradely via the aorta. Pig and sheep hearts were used in wedge preparations from the right ventricle (RV) section, perfused through the right coronary artery, and the edges of the cut-out section were clamped to maintain adequate coronary artery pressure. The pressure was maintained between 50 - 70 mmHg regulating the flow rate with a peristaltic pump in all hearts. Contraction motion was suppressed by (±)-Blebbistatin (Cayman Chemicals) at Tyrode's concentration of 2 - 3 μM, prepared as a stock solution dissolved in DMSO at the ratio of 5 mg/ml. V_m dye Di-4-ANBDQPQ (JPW-6003) (Potentiometric dyes) was previously prepared as a stock solution dissolved in ethanol at the ratio of 20 mg/ml. We used 0.05 mg of the V_m dye per one gram of tissue, approximately 0.5 mg of the V_m dye for whole cat and rabbit hearts, and 1 - 1.5 mg of the dye for wedge preparations of sheep and pig hearts. In the experimental setups to validate zero cross-talk, and to present the dual V_m - Ca measurement with a single camera, hearts were additionally stained with Rhod-2 $[Ca^{2+}]_i$ dye. $[Ca^{2+}]_i$ Rhod-2 dye (AAT Bioquest) was previously prepared as a stock solution dissolved in DMSO at the ratio of 1 mg/ml. We used 1 mg of the $[Ca^{2+}]_i$ dye for cat and rabbit hearts and 2 mg of the $[Ca^{2+}]_i$ dye for

pig and sheep heart preparations. The dyes were injected as a bolus in the cannulated aorta/right coronary artery over 3 minutes in small injection intervals 15 seconds apart, not interfering with the flow rate. The dye staining procedure of the Zebrafish hearts was done by incubating the hearts in oxygenated Tyrode's solution for 20 min containing 15 μM of the V_m dye and stimulated at the rate of 1 stimulus per second to induce contractions for better dye absorption. Afterwards, the hearts were additionally incubated for 30 min in 3 μM ($\pm$)-Blebbistatin.

Isolation and monolayer tissue culture of neonatal rat ventricular myocytes harvesting procedures followed the guidelines for federal research published by the National Institutes of Health and the protocol was approved by IACUC at Emory University. Neonatal rat ventricular myocytes (NRVM) were isolated from postnatal day two Sprague-Dawley rat pups (Charles Rivers) as previously described (Kapoor et al., 2013). Three-fourths of the ventricle were excised and treated with trypsin (1.25 mg/mL, Worthington Biochemical Corporation) overnight and then enzymatically treated with collagenase (1 mg/mL, Worthington Biochemical Corporation). Freshly isolated NRVMs were plated in the polystyrene cell culture dishes (2 x 10^6 cells/35 mm dish, Coring inc) which is coated in extracellular matrix protein, fibronectin (25 /mL, BS Bioscience) and cultured in M199 culture medium (Gibco) supplemented with 10% fetal bovine serum (FBS) (GE Healthcare), 3.5 mg/mL glucose (Sigma), 2 mM GlutaMAX, 100 U/mL penicillin, 4 μg/mL vitamin B12, 10 mM 4-(2-hydroxyethyl)-1-piperazineethanesulfonic acid (HEPES) buffer and 0.1 mM minimum essential medium (MEM) nonessential amino acids (Gibco). Cells were washed with Phosphate-buffered saline (PBS) and culture media was changed 24 hours after initial seeding. On day 2, culture media was replaced from 10% to 2% FBS and changed every other day. Optical mapping was performed on day 4 and 5 with 2% FBS culture media without phenol red.

EXCITATION LIGHT SOURCE CHARACTERIZATION

High power green LED of 525 nm nominal center wavelength (Luminous Devices), and red LEDs of 632 nm and 660 nm nominal center wavelengths (LEDEngin) were used as light sources for the V_m dye excitation, powered from a stabilized current source (PLUMBUS, donated from Aleksa Tech) (Figure 1C and Supplementary Figure 3). The LED light was collimated with the plano-convex lens (Thorlabs) to achieve zero angles of incidence with the thin-film based band pass (BP) excitation filters; as for the thin-film filters, the filter's passband cut-on wavelength depends on the angle incidence. Not addressing this relationship would lead to experimental errors and increased variability in the obtained measurements. The collimated light was first wideband filtered using 550/88 nm OD6 filter (Semrock) for the green LED or 650/100 OD6 filter for the red LEDs (Semrock) before filtered through a 10 nm wide BP OD4 filters. For $\Delta F/F$ measurement, hearts stained with the V_m dye were illuminated (excited) using a range of 10 nm wide BP filters, with the nominal center wavelengths at 500, 510, 520, 532, 540, 546, 550, 555, 560, 568, 580, 619, 632, 640, 650, 660, and 671 nm, with 2 nm center wavelength production tolerance (Edmund Optics).

The LED's emission spectra have a characteristic bell-shaped curve (Supplementary Figure 3), resulting in non-uniform intensity in the excitation filter's BP range. The center wavelength of a BP filter is not a valid measure as a single effective excitation wavelength, and in order to obtain a single effective excitation wavelength of each BP filter, the filtered excitation light's spectra were measured with a BP filter placed in the illumination setup (Figure 1C). With this approach, any non-zero angle of incidence is taken into account by performing the actual measurements of the transmitted light's spectral profile used for excitation in the experiments (Supplementary Figure 3). The spectra were measured across 200 points for each BP filter with a perpendicularly placed optical fiber (400 μm core) across the filter surface connected to a spectrometer (Ocean Optics) with a 10 μm wide entrance slit. The entrance slit width result in pixel

resolution of 3.2 pixels with spectrometer line sensor of 3648 pixels, achieving a spectral resolution of 0.25 nm across the spectrometer sensitivity range from 500 to 800 nm. The spectrometer was calibrated using the Hg–Ag lamp (Ocean Optics). For each BP filter, 200 measurements were averaged, and the effective excitation wavelength, (λ_{Eff}), was obtained as the mean wavelength of transmitted light intensity across the filter transmission band (Equation 1). $T(\lambda)$ is the transmitted light intensity, λ_{L}, and λ_{U} are the lower and upper wavelengths 20 nm less and 20 nm greater than the specified nominal center wavelength respectively.

$$\int_{\lambda_{\mathrm{L}}}^{\lambda_{\mathrm{Eff}}} T(\lambda) d\lambda = \frac{1}{2} \int_{\lambda_{\mathrm{L}}}^{\lambda_{\mathrm{U}}} T(\lambda) d\lambda \tag{1}$$

EMISSION FILTERS CHARACTERIZATION

Emitted fluorescence was passed through a thin-film interference type LPF with the nominal cut-on wavelengths (λ_{LPF}) of 700 nm (Chroma Technologies), 715, 740, and 775 nm (Omega Optical). $\Delta F/F$ Measurements were performed for each LPF placed between the lens (Pentax 8 - 48 mm f/1.0) and the camera sensor. Due to the large tolerances on the manufacturer specified cut-on wavelengths, the λ_{LPF} of each LPF was determined experimentally. Using a quartz tungsten halogen lamp (Thorlabs) as the light source, the light was first collimated, and the spectrum of the transmitted light was measured and averaged across 200 spatial locations as described in the section above. To account for non-linearity of each LPF transmission edge, the λ_{LPF} was obtained as the mean value of the LPF spectra integrated for wavelengths corresponding to 10% and 90% of the filter rising edge, relative to the peak of transmitted light $T(\lambda)$ (Equation 2). For each LPF, the measurements were normalized using the quartz lamp emission spectra profile (Supplementary Figure 2).

$$\int_{\lambda(10\%)}^{\lambda_{\mathrm{LPF}}} T(\lambda) d\lambda = \frac{1}{2} \int_{\lambda(10\%)}^{\lambda(90\%)} T(\lambda) d\lambda \tag{2}$$

ABSORPTION AND EMISSION SPECTRA OF V_M DYE JPW-6003

For a given V_m dye, absorption spectra depends on many parameters, such as dye solvents. The absorption spectra of JPW-6003 dye measured in ethanol, multilamellar vesicles or in hemispherical lipid bilayer (Pertsov et al., 2012, 2017; Matiukas et al., 2007), and also pH and Mg ATP (Clarke et al., 1992) resulting in significantly different absorption curves. When the dye is bounded to a cell membrane and subjected to the strong electric field across the membrane, the absorption spectra can be altered. The absorption spectra also depend on tissue absorbance, making it difficult to standardize the dye absorption spectra in tissue. Among these reasons, the absorption spectra of the JPW-6003 dye is simulated with readily available spectra of the Di-4-ANNEPS V_m dye obtained using Fluorescence Spectra Viewer (Thermo Fisher). The emission spectrum was shifted, aligning the spectra emission peak with the point of the JPW-6003 at 578 nm (Uzelac et al., 2019) (Supplementary Figure 1). Emission spectrum was measured in isolated Langerdorff perfused rabbit hearts, stained with JPW-6003 dye, across 200 points on the filter surface using perpendicularly placed optical fiber, connected to the spectrometer as in the section above. The long-pass-filter (LPF) of 575 nm nominal cut-on wavelength (Chroma) was used to decouple the excitation light. The tissue was illuminated using 10 nm wide BP excitation filter with the 540 nm nominal center wavelength. The fluorescence signal was integrated for 10 s, and multiple measurements were averaged.

The simulated absorption spectra and experimentally obtained emission spectra of the JPW-6003 are a good first approximation to hypothesize the range of semasbestic wavelengths and to describe the magnitude of $\Delta F/F$ as a function of the λ_{LPF} and λ_{Exc} (Supplementary Figure 1B,1E).

$\Delta F/F$ MEASUREMENT

The emitted fluorescence intensity F, detected by the camera, is a function of the V_m dye absorption coefficient A, emission spectra integrated over the range of wavelengths passed through the LPF with a cut-on wavelength λ_{LPF}, and the camera sensor's spectral sensitivity S (Equation 3). Additionally, the absorption coefficient depends on the excitation wavelength λ_{Exc}, where the absorption spectra shift is approximated linearly in V_m. For a given camera and V_m dye, the maximal fractional change of fluorescence depends on the choice of the excitation wavelength and the LPF (Equation 4).

$$F\left(\lambda_{\text{Exc}}, \lambda_{\text{LPF}}, V_m(t)\right) = \int_{\lambda_{\text{LPF}}}^{\infty} S(\lambda) \cdot A(\lambda_{\text{Exc}} + kV_m(t)) \cdot E(\lambda + kV_m(t)) d\lambda \tag{3}$$

$$\frac{\Delta F\text{max}}{F} = \left| \frac{F(\lambda_{\text{Exc}}, \lambda_{\text{LPF}}, V_m^{\text{max}}) - F(\lambda_{\text{Exc}}, \lambda_{\text{LPF}}, V_m^{\text{min}})}{F(\lambda_{\text{Exc}}, \lambda_{\text{LPF}}, V_m^{\text{min}})} \right| \tag{4}$$

In the experimental measurements of the fractional fluorescence change, $\Delta F/F$, each λ_{Exc} term represents a single excitation wavelength equal to the λ_{Eff} wavelength of a 10 nm wide BP excitation filter, as described above. The λ_{Eff}, divides the excitation light spectra into two sub-spectra of equal light intensities, and $\Delta F/F$ dependence on the excitation wavelength can be approximated as linear in the 10 nm narrow range. As a result, $\Delta F/F$ increase in the sub-spectra left of the λ_{Eff} will be equal to $\Delta F/F$ decrease right of the λ_{Eff}. Based on this reasoning, the excitation band for a given 10 nm BP filter can be approximated with a single effective excitation wavelength λ_{Eff}. Additionally, the $\Delta F/F$ change is less than 0.05% per 1 nm change of the excitation wavelength, thus minimizing any experimental error with this first-order approximation (Figure 2D).

Fluorescence signals were acquired as the sequence of images using EMCCD camera (Evolve 128, Photometrics) at the resolution of 64 x 64 (2 x 2 binning) or 128 x 128 pixels, digitized at 16-bits, and transferred to a PC via real-time uninterrupted data transfer. A custom acquisition/camera control program was used. V_m signals were acquired at 500 fps, while in experimental setups with hearts stained with both V_m and $[Ca^{2+}]_i$ dye, each signal was acquired at 250 fps. To use a single camera to measure two fluorescence signals (i.e. bi-modal), alternating frames record each dye's signals. The fluorescence dyes are individually excited with separate light sources of different wavelengths, alternating synchronously with the camera frame rate. A readout signal from the camera was used as a reference clock to a microcontroller to generate a trigger signal at pre-defined intervals to feed into a heart simulator (World Precision Instruments) for subsequent data ensemble averaging (stacking). The stimulating current was adjusted to be twice the stimulating threshold, 3 – 5 mA.

The recording protocol was defined as follows: In the preliminary experiments using isolated rabbit hearts, for each of the four LPFs, the entire range of BP excitation filters from 500 to 671 nm was used. In all 31 experiments, hearts were equilibrated and paced at a constant stimulation period until a steady state was reached. The pacing period was 300 - 500 ms for all animals, except for fish hearts of 700 ms. For each LPF-BP pair, hearts were paced for the duration between 2 - 4 minutes, except for fish hearts. The longer

duration was used for near the *semasbestic* wavelengths. Based on the preliminary experiments, *semasbestic* wavelengths were determined in the range between 520 and 560 nm. In subsequent measurements on other isolated hearts, only the green LED was used with the excitation filters ranging from 500 to 580 nm, as for each isolated heart the experimental protocol requires steady-state conditions often exceeding 4 hours to perform recordings for all LPF-BP filter pairs. In setups with fish hearts, recordings were longer and up to 10 minutes for excitation near the *semasbestic* points, due to small preparations resulting in more noisy signals (Supplementary Figure 4). Due to long required times to perform recordings for all filter combinations, in some heart preparation, due to heart conditions degradation, the measurements were stopped before all four LPF were exchanged, resulting in different number of measurements for each animal heart (Figure 2B)

SIGNAL AND IMAGE PROCESSING

Post-processing and data analysis was performed in MATLAB and a custom-developed Java applet.

Baseline subtraction. A level of 500 counts was subtracted from optical mapping signals corresponding to each pixel (pixel trace), as the EMCCD camera adds 500 counts to each A/D converted value. For each pixel trace, the baseline was obtained passing the signal through a low-pass Kaiser window filter of the passband from 0 Hz to 1/2 of the pacing frequency, and the stopband greater than the pacing frequency, with 40 dB attenuation between the passband and the stopband, and 0.1 dB allowed ripple in the passband. The obtained baseline was subtracted, and the result was divided by the baseline. The procedure was performed for each pixel trace, obtaining fractional fluorescence change over time ($\Delta F/F$).

Ensemble averaging (stacking). Each recording typically lasted between 2 and 4 minutes, and up to 10 minutes for fish hearts. Longer recordings were used for noisy signals corresponding to small $\Delta F/F$ changes when the excitation wavelength is close to the *semasbestic* wavelength, making the exact determination of the $\Delta F/F$ magnitude difficult without filtering. To avoid filtering, which would result in smoothed signal amplitudes and experimental errors, stacking Uzelac and Fenton (2015) was performed, averaging 200 to 500 action potentials (APs) and up to 1000 APs for fish hearts. The stacking method significantly improved the signal-to-noise ratio (SNR) (Figure 1C and Supplementary Figure 4).

Photobleaching. As with any optical mapping recordings, $\Delta F/F$ typically decreases over time due to photobleaching and dye internalization. With required long recording times, in order to compensate for the $\Delta F/F$ dependence over time, approximately every 20 minutes, a control measurement was performed using LPF of λ_{Eff} = 700 nm and excitation using 660 nm centered BP filter, to measure $\Delta F/F$ changes from the beginning of the experiment. From the measurements, a curve was fitted to calculate correction factors for each $\Delta F/F$ value corresponding to each LPF-BP filter pair.

Classification of AP signals. In the optical mapping experiments, mechanical heart contractions were decoupled with ($\pm$)-Blebbistatin. However, even with the decoupler, minuscule contractions can be observed for low amplitude $\Delta F/F$ signals, as with excitation near the *semasbestic* wavelength. In the post-processing, all pixels were cross-correlated, and only pixels with a correlation coefficient greater than 0.97 were selected, while the others were discarded. Typically, discarded pixels represented less than 5% of the total imaged heart surface. For each stacked AP signal, SNR was calculated as the ratio of its $\Delta F/F$ amplitude over the AP segment's root-mean-square value during the flat repolarization phase. Stacked APs with SNR < 10 were discarded. Stacked APs of SNR between 10 and 30 were additionally filtered with an adaptive anisotropic 3-D diffusion filter, and the SNR value was recalculated. The procedure was repeated up to three times. If SNR was not greater than 30, such stacked APs were discarded. Typically,

for unfiltered stacked APs, SNR values ranged from 50 to 300 depending on proximity to the *semasbestic* wavelengths.

$\Delta F/F$ calculation and determination of semasbestic wavelengths. For each experiment and each LPF-BP filter pair, from selected APs the mean $\Delta F/F$ and its standard deviation were calculated. In each experiment and for each LPF, a linear or quadratic polynomial curve were fitted for different BP values (Figure 1D). The fit's choice was based on the R^2 value of the fit, and fitting was performed as a weighted fit. The standard deviation of $\Delta F/F$ values are taken as y-uncertainties. X-uncertainties (excitation wavelengths) were estimated from y-uncertainties from partial derivatives (slope) of $\Delta F/F$ non-weighted fit curves and summed in squares with the excitation wavelength uncertainties (Supplementary Figure 3). Typically, y-uncertainties were the order of magnitude larger. *Semasbestic* wavelength for each LPF in each experiment (heart) was obtained as zero crossings of the fitted curve (Figure 1F).

Statistical Analysis

Descriptive data are presented as absolute numbers, percentages or means, with respective one standard deviation, unless noted otherwise. A one-way ANOVA test was used to determine significance between the experiment on different animal species. *P* values less than 0.05 were considered significant. Analyses were conducted using MATLAB software (version 2020, Mathworks)

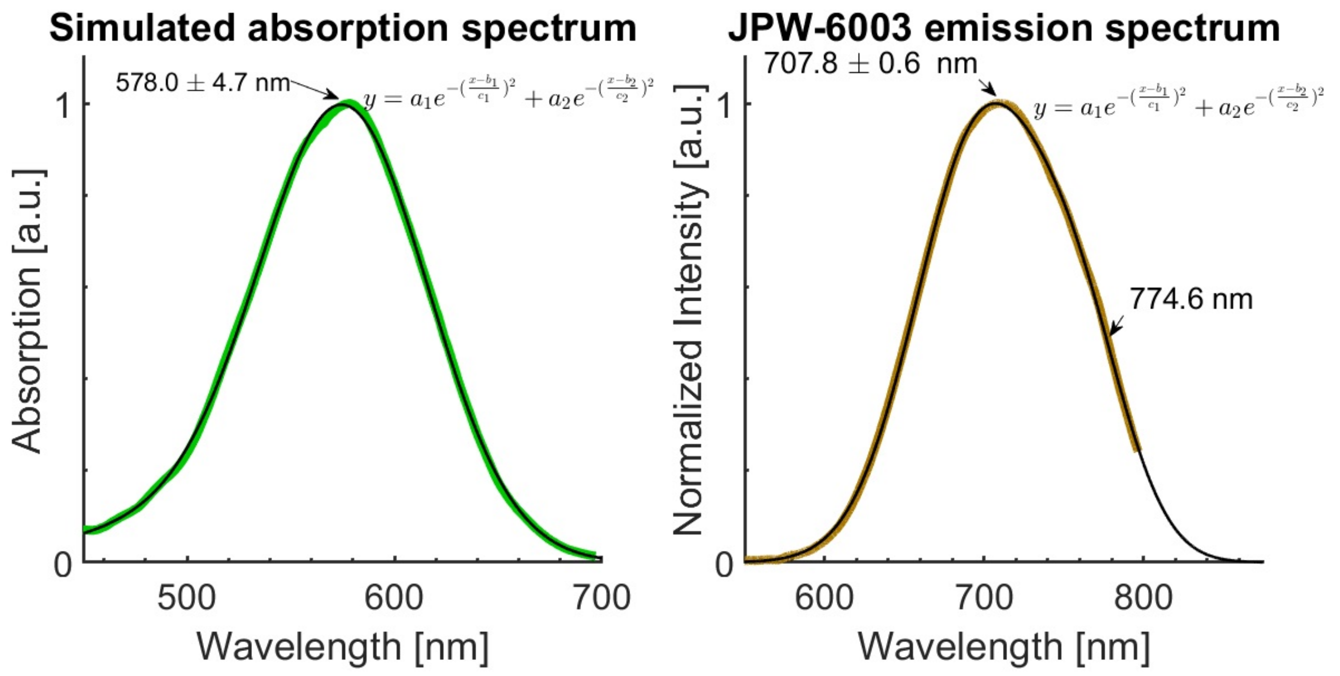

Supplementary Figure 1. Absorption and emission fluorescence spectra of V_m JPW-6003 dye. Two term Gaussian curves were fitted for both absorption and emission spectrum with the following coefficients expressed with 95% confidence bounds: a_1 = 0.958 (0.827, 1.09), b_1 = 0.0484 (0.0289, 0.0679), c_1 = 0.794 (0.771, 0.817) , a_2 = 0.0832 (0.0272, 0.139), b_2 = -0.962 (-2.016, 0.091) , c_2 = 1.145 (0.292, 2.000). Parameters for the experimentally obtained emission spectra are as follows: a_1 = -1.47 (-1.77, -1.17), b_1 = 0.518 (0.515, 0.521), c_1 = 0.713 (0.702, 0.725), a_2 = 2.45 (2.15, 2.75), b_2 = 0.488 (0.486, 0.491), c_2 = 0.839 (0.831, 0.847). As shown, the absorption spectrum peak is centered at the isosbestic point of 578.0 ± 4.7 nm. The emission spectrum peak is at 707.8 ± 0.6 nm and 50% emission at 774.6 nm.

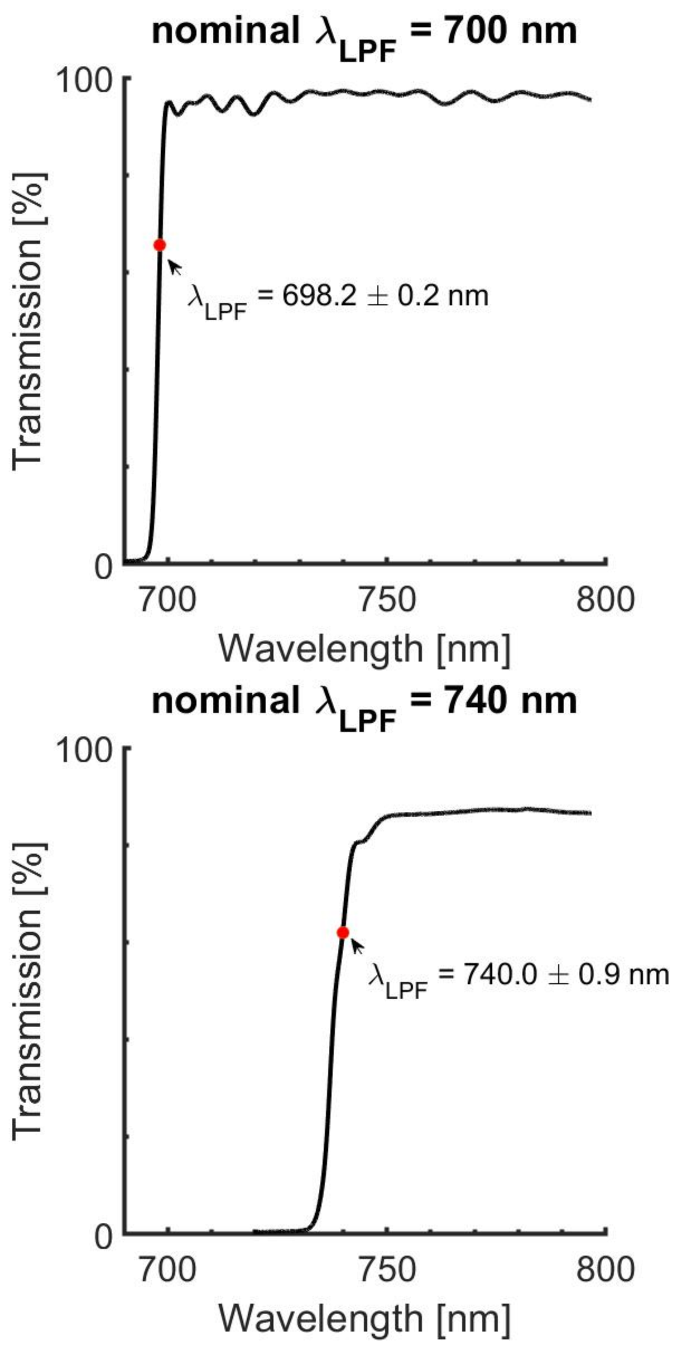

Supplementary Figure 2. Transmission spectra of LPFs with given manufacturer specified nominal and measured cut-on wavelengths λ_{LPF}. The measurements were performed integrating the transmission curve rising from 10% to 90% in amplitude relative to the transmission peak, representing the total integral value. The λ_{LPF} is determined as the wavelength at which the integral value starting at 10% amplitude relative to the transmission peak reaches 50% compared to the total integral value.

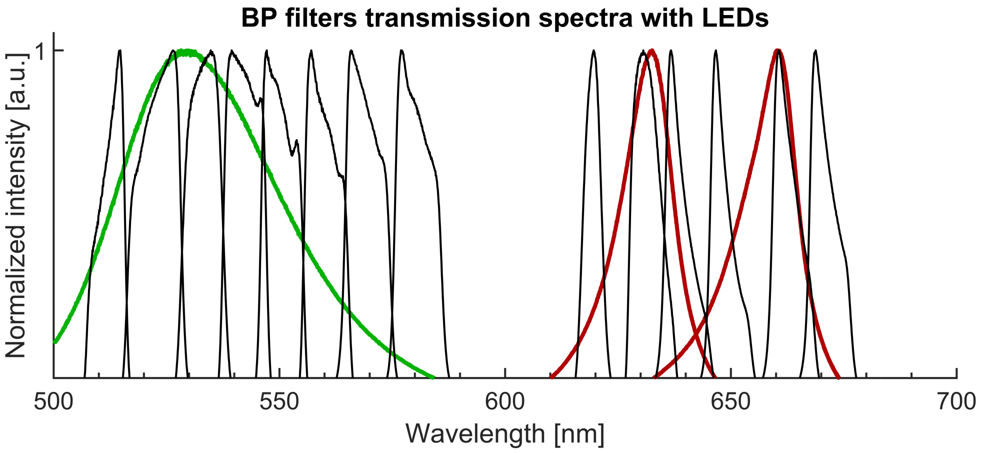

λ_{Nom} [nm]	510	520	532	540	546	550	555	560	568	580
λ_{Eff} [nm]	510.9 ± 0.9	521.4 ± 0.7	532.1 ± 0.7	541.5 ± 0.7	546.9 ± 0.4	550.0 ± 0.4	558.1 ± 0.3	559.9 ± 0.3	568.3 ± 0.3	579.5 ± 0.2

λ_{Nom} [nm]	619	632	640	650	660	671
λ_{Eff} [nm]	619.4 ± 0.3	631.2 ± 0.7	638.3 ± 0.4	648.0 ± 0.3	658.2 ± 0.4	670.6 ± 0.3

Supplementary Figure 3. Measured LEDs spectrum and BP filters transmission spectrum coupled with the LEDs.. For $\Delta F/F$ measurement with different excitation bands three different LEDs were used in the optical mapping experiments, given with the nominal manufacturer specified peak wavelengths at 525 nm (green, Luminous Devices), 632 nm (red, LedEngin) and 660 nm (red, LedEngin). Each 10 nm wide BP filter is specified with the nominal manufacturer specified center wavelength λ_{Nom} with $\pm$ 2 nm production uncertainty. The effective excitation (center) wavelength, λ_{Eff} was measured for BP filters of λ_{Nom} from 510 to 580 nm coupled with 525 nm peak LED, λ_{Nom} from 619 to 640 nm coupled with 632 nm peak LED, and λ_{Nom} from 650 to 671 nm coupled with 660 nm peak LED. The nominal and measured effective values of the BP filters are shown in the table. Due to spectrometer lower range limit of 500 nm, the λ_{Eff} of the 10 nm wide 500 nm centered BP filter was not measured.

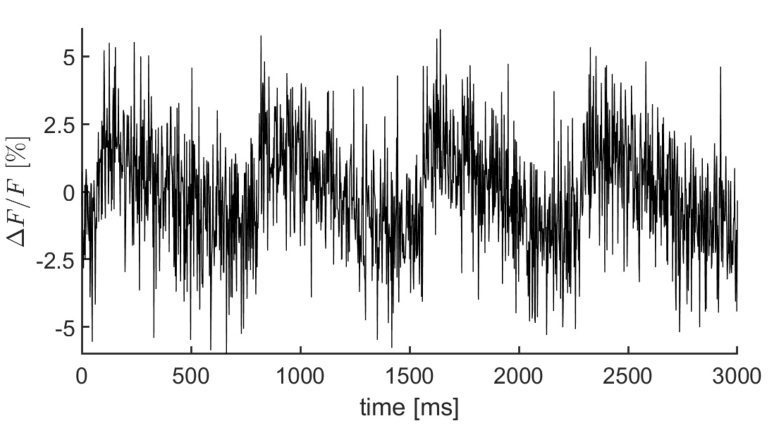

Supplementary Figure 4. The effect of stacking. In small heart preparations such as from zebra-fish, AP signals of low SNR make it difficult to accurately obtain the amplitude of the AP, the $\Delta F/F$ magnitude. Averaging (stacking) over 500 individual APs, the SNR is substantially improved, and $\Delta F/F$ can be obtained without the need for additional signal filtering which would otherwise smooth the signal and affect the $\Delta F/F$ magnitude. To apply the stacking procedure, up to 500 APs were recorded at a pacing cycle length of 700 ms, requiring a recording duration exceeding 10 minutes for each LPF-BP pair.

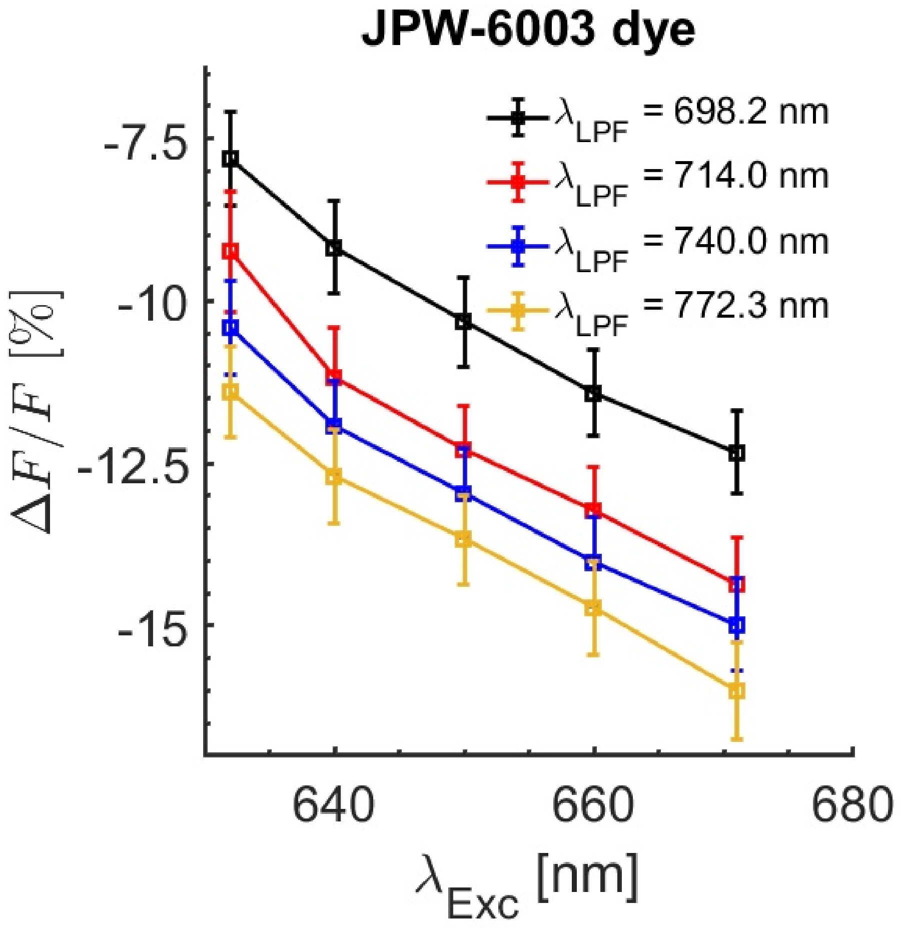

Supplementary Figure 5. $\Delta F/F$ **magnitude values for** V_m **JPW-6003 dye as a function of** $\lambda_{\mathbf{LPF}}$ **and** $\lambda_{\mathbf{Exc}}$**.** Isolated rabbit heart was stained with the JPW-6003 dye and illuminated using different 10 nm wide BP filters of the nominal center wavelengths of 632, 640, 650, 660 and 671 nm. For each BP-LPF filter combination $\Delta F/F$ was obtained as average across the imaged side of the heart. The total length of each error bar is two standard deviations. With longer λ_{Exc} excitation wavelengths and using LPFs of longer λ_{LPF}, $\Delta F/F$ increases in magnitude. Shown λ_{Exc} values are effective experimentally measured cut-on wavelengths of the LPFs.

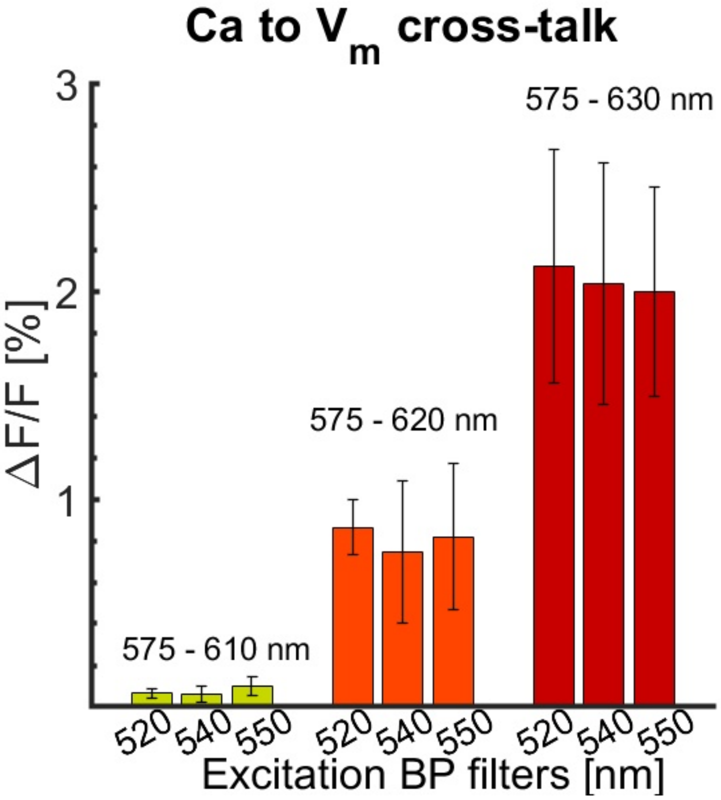

Supplementary Figure 6. Ca dye excitation and V_m dye induced cross-talk. Guinea pig heart was stained with the JPW-6003 dye and illuminated using different 10 nm wide BP filters of nominal center wavelengths of 520, 540, and 550 nm. For each BP filter, $\Delta F/F$ was obtained for three different emission bands, 575-610, 575-620 and 575-630 nm. The bands were selected using OD6 LPF of 575 nm nominal wavelength (Chroma) and three different OD6 short-pass filters of the nominal wavelengths of 610, 620 and 630 nm (Omega Optical) on the camera side. The shown $\Delta F/F$ values were obtained as averages across the imaged side of the heart. The total length of each error bar is two standard deviations. Each bar represents the amount of cross-talk, the V_m signal in the emission band of a given Ca dye. From the obtained $\Delta F/F$ measurements, the upper limit of the Ca emission band has to be no more than 610 nm.

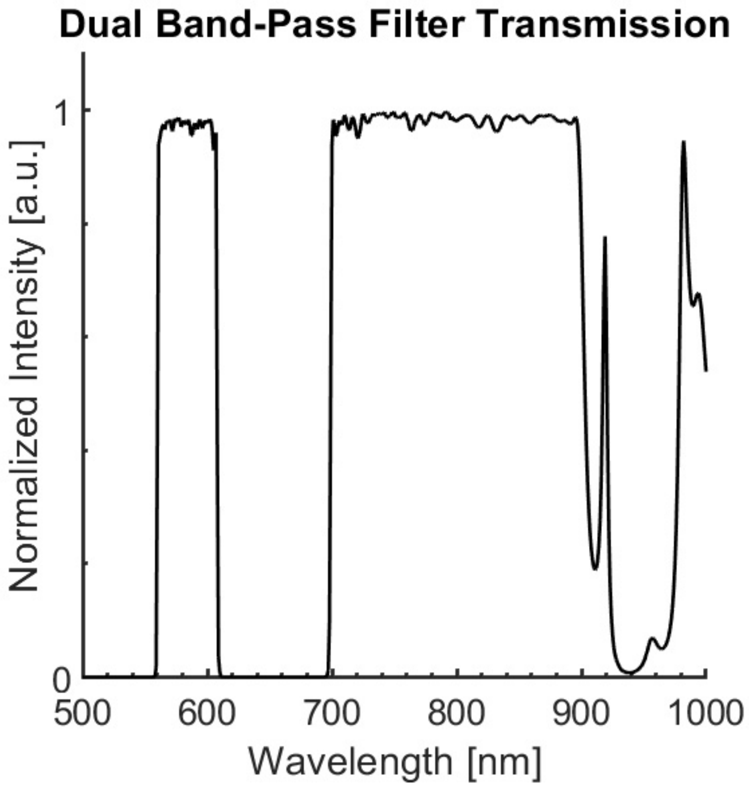

Supplementary Figure 7. The transmission spectra of the dual band-pass filter The first passband is in the nominal range of 560 - 610 nm, and the second LPF band starts from the nominal 700 nm wavelength (measured 698.2 nm). The filter spectra were provided by the manufacturer (Chroma).

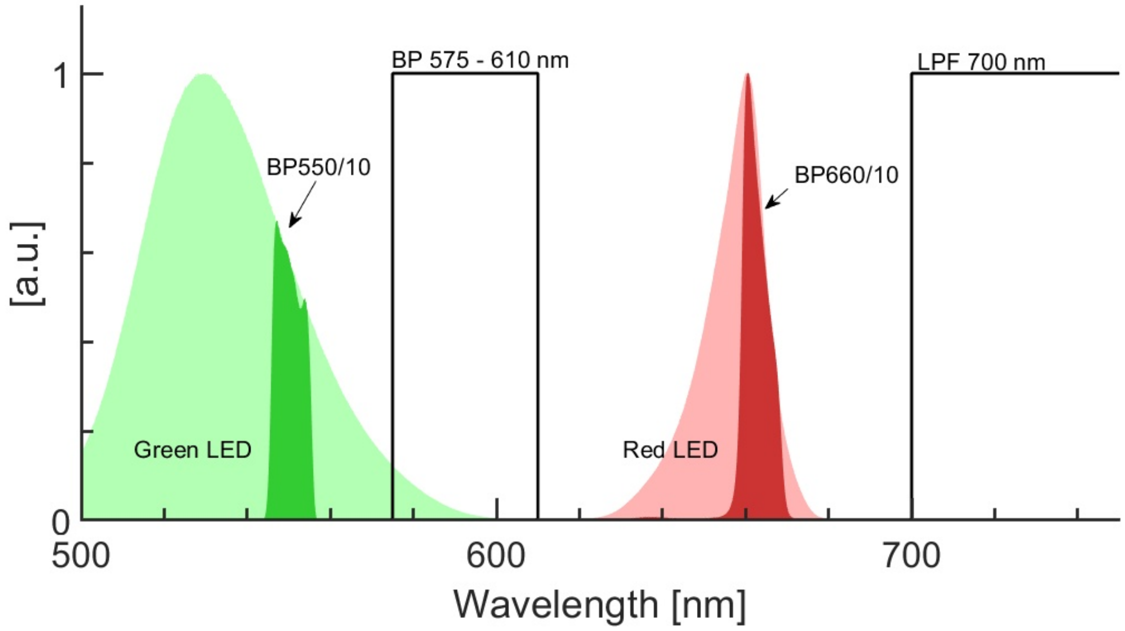

Supplementary Figure 8. Illustration of the excitation and emission filters spectra for optical mapping setup using off-the-shelf filters. JPW-6003 V_{m} was excited using the red LED of 660 nm center wavelength coupled with the 10 nm wide BP filter of 660 nm nominal center wavelength. The illustration shows the actual light band spectra measured with the red LED passing through the BP660/10 filter. Emitted V_{m} fluorescence is passed through the LPF 700 nm filter on the camera side. Rhod-2 Ca was excited using the green LED of 525 nm center wavelength coupled with the 10 nm wide BP filter of 550 nm nominal center wavelength. The illustration shows the actual light band spectra measured with the red LED passing through the BP550/10 filter. Emitted Ca fluorescence is passed through the dual BP filter on the camera side of the first band from 560 to 610 nm, with an additional LPF of 575 nm nominal cut-on wavelength to allow enough separation from the Ca dye excitation filter band.

Supplementary Video 1. Simultaneous measurement of propagating AP across isolated rabbit hearts with JPW-6003 and Rhod-2 fluorescent dyes. Data were obtained at fast pacing cycle lengths from 146 to 200 ms and presented separately for each cycle length and separately for V_m and Ca signals, for two consecutive beats. The correction was made interpolating time series for pacing cycle lengths under 200 ms in order for each cycle length to last the same time duration for visual comparison of propagation speed and repolarization rates across different cycle lengths. For cycle lengths under 160 ms, large spatial repolarization gradients are formed in Ca in the heart's lower segment. As a result, spatially discordant alternans in V_m signal (spatial regions of different repolarization times) are formed, slowing down propagation of the AP and prolonging AP duration as shown with increased repolarization time (the appearance of yellowish regions at cycle lengths under 160 ms compared to the cycle length of 200 ms). Spatially discordant alternans lead to the formation of T-wave alternans on ECGs, a well-known marker for arrhythmia susceptibility.

Supplementary Video 2. Simultaneous measurement of propagating AP across isolated rabbit hearts with JPW-6003 and Rhod-2 fluorescent dyes. Data were obtained at a range of pacing cycle lengths from 136 to 350 ms and presented separately for each cycle length, V_m and Ca signals, and separately for even and odd beats. The correction was made interpolating time series for pacing cycle lengths under 350 ms in order for each cycle length to last the same time duration for visual comparison of propagation speed and repolarization rates across different cycle lengths. Large repolarization gradients in both Vm and Ca signal are present at physiological rated for rabbit, for cycle lengths above 200 ms, induced by inadequate heart tissue perfusion rate.

REFERENCES

Clarke, R. J., Schrimpf, P., and Schöneich, M. (1992). Spectroscopic investigations of the potential-sensitive membrane probe rh421. *Biochimica et Biophysica Acta (BBA)-Biomembranes* 1112, 142–152

Kapoor, N., Liang, W., Marbán, E., and Cho, H. C. (2013). Direct conversion of quiescent cardiomyocytes to pacemaker cells by expression of tbx18. *Nature biotechnology* 31, 54–62

Matiukas, A., Mitrea, B. G., Qin, M., Pertsov, A. M., Shvedko, A. G., Warren, M. D., et al. (2007). Near-infrared voltage-sensitive fluorescent dyes optimized for optical mapping in blood-perfused myocardium. *Heart rhythm* 4, 1441–1451

Pertsov, A. M., Matiukas, A., Loew, L. M., and Wuskell, J. P. (2012). Composition, method, system, and kit for optical electrophysiology US Patent 8,155,730

Pertsov, A. M., Matiukas, A., Loew, L. M., and Wuskell, J. P. (2017). Composition, method, system and kit for optical electrophysiology US Patent 9,636,424

Uzelac, I., Crowley, C. J., and Fenton, F. H. (2019). Isosbestic point in optical mapping; theoretical and experimental determination with di-4-anbdqpq transmembrane voltage sensitive dye. In *2019 Computing in Cardiology Conference (CinC), Singapore* (IEEE), Page–1

Uzelac, I. and Fenton, F. H. (2015). Robust framework for quantitative analysis of optical mapping signals without filtering. In *2015 Computing in Cardiology Conference (CinC), Nice* (IEEE), 461–464
